# Neurophysiological assessment of biometric patterns during semi-immersive and traditional learning experiences in the humanities

**DOI:** 10.3389/fnhum.2026.1692599

**Published:** 2026-02-03

**Authors:** Rebeca Romo-De León, Mei Li L. Cham-Pérez, Verónica Andrea Elizondo-Villegas, Alejandro Villarreal-Villarreal, Alexandro Antonio Ortiz-Espinoza, Carol Stefany Vélez-Saboyá, Jorge de Jesús Lozoya-Santos, Manuel Cebral-Loureda, Mauricio A. Ramírez-Moreno

**Affiliations:** 1 Mechatronics Department, School of Engineering and Sciences, Tecnologico de Monterrey, Monterrey, Mexico; 2 School of Humanities and Education, Tecnologico de Monterrey, Monterrey, Mexico

**Keywords:** electroencephalography, emotions, immersive technologies, learning environments, neurohumanities

## Abstract

The use of immersive technologies in education has shown an improvement in the learning process of students. However, applications of these technologies in the Humanities are limited, since most studies focus on scientific fields. In this study, the Neurohumanities Lab was introduced as a semi-immersive space for teaching the Humanities. Two groups of 12 participants each performed activities under the semi-immersive and traditional classroom set-ups, while recording their physiological signals (electroencephalography, electrodermal activity, and heart rate). In both groups, the ITC-SOPI presence questionnaire was used to compare their differences in perceived presence levels, which showed a higher level in the experimental group. Machine learning algorithms were applied, concluding that the decision tree supervised learning model determined the most relevant features to distinguish between both set-ups with an accuracy of 90%. In the experimental group, an increased heart rate was observed with respect to the control group, while the electrodermal activity increased its peaks in both groups compared to the basal state. Additionally, brain source localization techniques revealed a notorious activation of brain areas related to emotional and somatosensory processing during the semi-immersive experience. Therefore, the Neurohumanities Lab has the potential to be a fully immersive environment for innovative education and enhanced learning.

## 1 Introduction

The Humanities encompass all aspects of human society and culture, including language, literature, philosophy, law, politics, religion, art, history, and social psychology. They offer insights into human evolution and learning (Carew and Ramaswami, 2020). Educational innovation has predominantly focused on science and engineering, where technological advancements have demonstrably enhanced learning outcomes (Bond et al., 2020). Conversely, the arts and Humanities have experienced comparatively little empirical investigation into novel pedagogical approaches (Hutson and Olsen, 2021). Nevertheless, the Humanities foster critical thinking, empathy, and creativity competencies which are crucial for innovation across all domains (Hutson and Olsen, 2021). Therefore, it is important to improve pedagogical methods to engage students more effectively in disciplines not only related to sciences but also to the Humanities.

The incorporation of technology in education has the potential to enhance learning and engagement in students, although it is crucial to have careful planning and use appropriate tools (Bond et al., 2020; Pradeep et al., 2024). Some of the educational technology tools used are online learning platforms, discussion boards, knowledge organization, videos, digital games and blogs (Bond et al., 2020; Bedenlier et al., 2020; Pradeep et al., 2024). Virtual reality and immersive learning spaces are other innovative educational technologies that show promise in the design of an engaging learning environment that could enhance the learning experience (Collins et al., 2019; Liu et al., 2024). There has been great interest in studying the influence an environment or tool has on a person's cognitive state especially in education (Gramouseni et al., 2023).

Neuroeducation is a topic that has gathered interest in recent years, considering that it seeks to understand how the brain operates in learning environments (Jolles and Jolles, 2021). It is the crossover between education and neuroscience, this field could inform better decision-making related to learning environments, improve learning outcomes, and pedagogical methods (Jolles and Jolles, 2021; Pradeep et al., 2024). Tools have been sought and developed for the evaluation of educational technologies, which have been mainly based on likert-scaled questionnaires that still deliver subjective information. The most widely used questionnaires for this evaluation are closely related to presence, as an indicator of a person's engagement, learning, and emotional reaction to the learning process (Ochs and Sonderegger, 2022). In literature reviews, presence has been studied as an important factor in understanding immersion and the effectiveness of virtual reality environments (Moinnereau et al., 2022; Grassini and Laumann, 2020). Most studies do not use a direct approach in evaluating presence and virtual environments in educational settings, which limits its evaluation and continuous improvement. Moreover, a quantitative measurement of the impact of virtual and immersive environments in educational set-ups may improve educational innovation applications.

Neurotechnology such as Functional Magnetic Resonance (fMRI), Positron Emission Tomography (PET), and Electroencephalography (EEG) have greatly contributed to a better understanding of how the brain operates in learning environments (Pradeep et al., 2024). EEG is one of the most common signals used to obtain important information on neural activity, which has been shown to reflect cognitive performance and emotional functions (Gramouseni et al., 2023; Cardona-Álvarez et al., 2023). Electrodermal activity (EDA) and blood volume pressure (BVP) are other physiological signals that can provide important information on the state of a subject's physiological functions and can be used as a measurement of a subject's mental state (Cosoli et al., 2021; Khedher et al., 2019). EDA has been considered a measure of the sympathetic nervous system, a branch of the autonomic nervous system, as it measures the variations in skin conductance (Cosoli et al., 2021; Goussain et al., 2025). EDA can be decomposed into tonic and phasic components, representing the slow and fast variations of EDA respectively (Veeranki et al., 2024). The phasic component is represented by the skin conductance response (SCR) and is commonly studied due to their correlation with cognitive activity (Moinnereau et al., 2022; Rahma et al., 2022). BVP measures changes of blood volume and has been identified as a tool for assessing engagement in educational environments (Moinnereau et al., 2022; Goussain et al., 2025). By understanding the subject's mental state, the learning systems can be adjusted to enhance learning outcomes.

The biometrics mentioned can evaluate patterns that correlate with sense of presence and engagement. EEG signals are most commonly studied in five frequency bands: delta (0.5–4 Hz), theta (4-8 Hz), alpha (8–12 Hz), beta (12-30 Hz), and gamma (>30 Hz) (Gramouseni et al., 2023). Some studies have assessed specific functions of the cognitive state in virtual reality based on EEG. For instance, a study analyzing a driving simulator found a higher cognitive load and a higher number of brain activation regions in virtual reality environments (Qadir et al., 2019). Another study involving storytelling in virtual reality provided evidence that participants sustained high levels of immersion and engagement noting an increase in alpha and theta band power (Škola et al., 2020). Studies related to EEG measurements and presence in immersive environments show a correlation with low power in all frequency bands in frontal electrodes (Moinnereau et al., 2022). Similarly, a high engagement index (obtained from EEG) indicates a higher immersive perception, and therefore, this neuromarker is a potential indicator of optimized learning in virtual environments (Ochs and Sonderegger, 2022; Moinnereau et al., 2022).

Specifically in the field of the Humanities, some new initiatives include digital Humanities and Neurohumanities, which try to create a significant and meaningful impact to the way these topics are taught today (Carew and Ramaswami, 2020). The interdisciplinary approach of neuroscience and Humanities is important, since it may enable linguistics, music, and emotional analysis to become key components of the understanding of modern cognitive neuroscience (Hartley and Poeppel, 2020). By combining virtual environments, digital Humanities and Neurohumanities, universities worldwide have implemented these ideas in subjects such as Art History and History of Architecture (Hutson and Olsen, 2021). However, they are still in the development stage, and questions such as measuring the real impact and benefit in learning outcomes from these technologies have arisen (Hutson and Olsen, 2021). Humanities studies are subjective, since learning is not quantitatively measurable, and answers to questions are not correct or incorrect as taught in exact sciences related subjects. Therefore, assessing learning outcomes in the Humanities becomes a challenge.

The arising questions and combination of Neuroscience and the Humanities, led to the Neurohumanities Lab (NH Lab) project, which proposes a design for a partially-immersive interactive system for the education of the Humanities. The system can detect movements, voice, facial gestures, emotions, and mental states of the user, with the use of cameras and wearable biometric devices. The emotional state of individuals were identified and analyzed with EEG, EDA, BVP, and temperature signals, along with computer vision techniques. The detected signals were used as a feedback loop to modify the participants environment, such as dynamic visualizations, lighting, and sound, allowing an enhanced environment-participant interaction (Blanco-Rios et al., 2024).

This approach provides individuals with the opportunity to interact with their environment and enhance their learning experience within a classroom setting. The underlying assumption is that by integrating these technologies, the learning experience can be improved in terms of engagement, personalization, multi-sensory stimulation, and overall effectiveness. The aim of the present study is to further evaluate the effectiveness of the NHLab design, by analyzing the demonstrated sense of presence and engagement in two groups of higher education students while completing four different activities, one group in a controlled traditional classroom setup and the other in the partially-immersive environment. Additionally, we aim to find which features in EEG signals can be used to differentiate between the experimental and control groups and whether EDA signals and heart rate variability can be used to determine which group experienced higher presence levels.

## 2 Methodology

### 2.1 Participants

The methodolog y for this research builds upon previous work (Romo-De León et al., 2024), in which the data collection process is described. The Dataset comprises a total of 24 Spanish-speaking adults between 18 and 25 years of age who were not undergoing medical treatment for any mental condition, which served as the inclusion criteria. All participants provided informed consent, confirming the approval of this study by the institution's ethics committee, the “Comité Institucional de Ética en Investigación (CIEI) del Instituto Tecnológico y de Estudios Superiores de Monterrey,” under the identification code EHE-2023-03.

### 2.2 Experimental protocol

Participants were divided into two groups: 12 participants in the control group and 12 participants in the experimental group. The objective of the experimental design was for both groups to complete four similar tasks, adapted to their respective set-ups: a traditional classroom and the NHLab. Tasks in both set-ups were based on a humanistic topic related to the book The Passions of the Soul by Descartes, an influential philosopher who contributed to the early conceptualization of emotions as they are understood today. Participants selected one of six Passions described by Descartes to represent and keep in mind throughout the tasks: admiration, love, hate, desire, joy, or sadness (Descartes, 2010).

**Figure 1:**
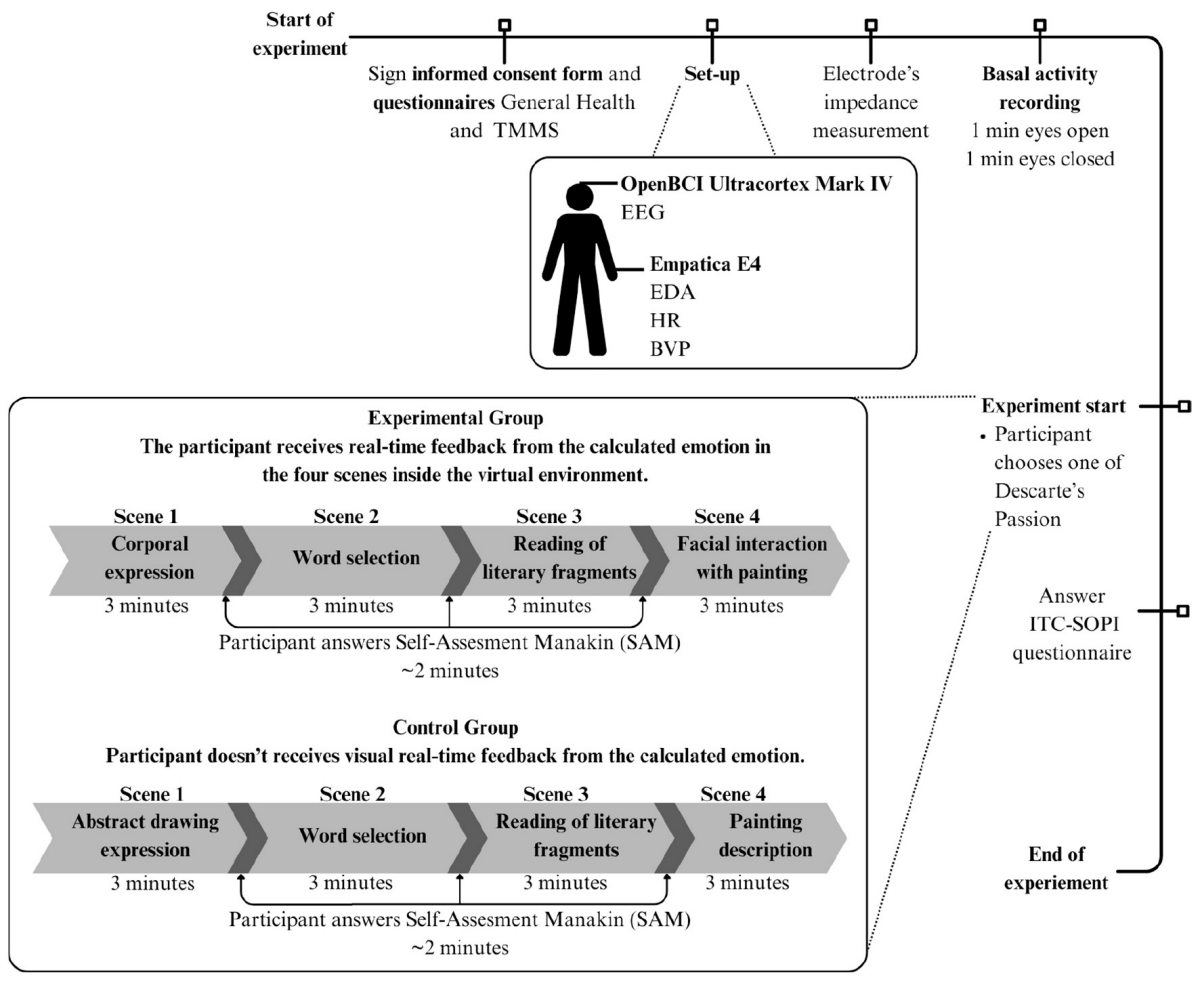
Methodology overview of the experimental protocol used for both groups. Diagram shows the process from start (**top left**) to end (**bottom right**).

A total of four questionnaires were completed by participants. Two were administered at the beginning of the experiment: the General Health Questionnaire (GHQ) and the Trait Meta-Mood Scale (TMMS-24) for emotional intelligence. The Self-Assessment Manikin (SAM) questionnaire was completed between each task, and the ITC-SOPI Presence questionnaire was administered at the end of the four tasks (Goldberg and Hillier, 1979; Bradley and Lang, 1994; Lessiter et al., 2001; Salovey et al., 1995). Biometric signal recording was conducted throughout the four tasks in both set-ups. Participants wore the OpenBCI Ultracortex IV helmet with eight electrode configurations (Fp1, Fp2, C3, C4, P7, P8, O1, O2), following the 10-20 International System for EEG recordings (OpenBCI, 2023). Additionally, an Empatica E4 wristband was used to record blood volume pulse (BVP), interbeat interval (IBI), electrodermal activity (EDA), and temperature (Empatica, n.d.). The overall methodology is illustrated in Figure 1.

#### 2.2.1 Scene description

During the experiment, the tasks were referred to as scenes, and each scene consisted of a 3-minute task. The experimental group was introduced to the NHLab, whereas the control group remained in a controlled classroom set-up consisting of a chair and a desk.

**Figure 2:**
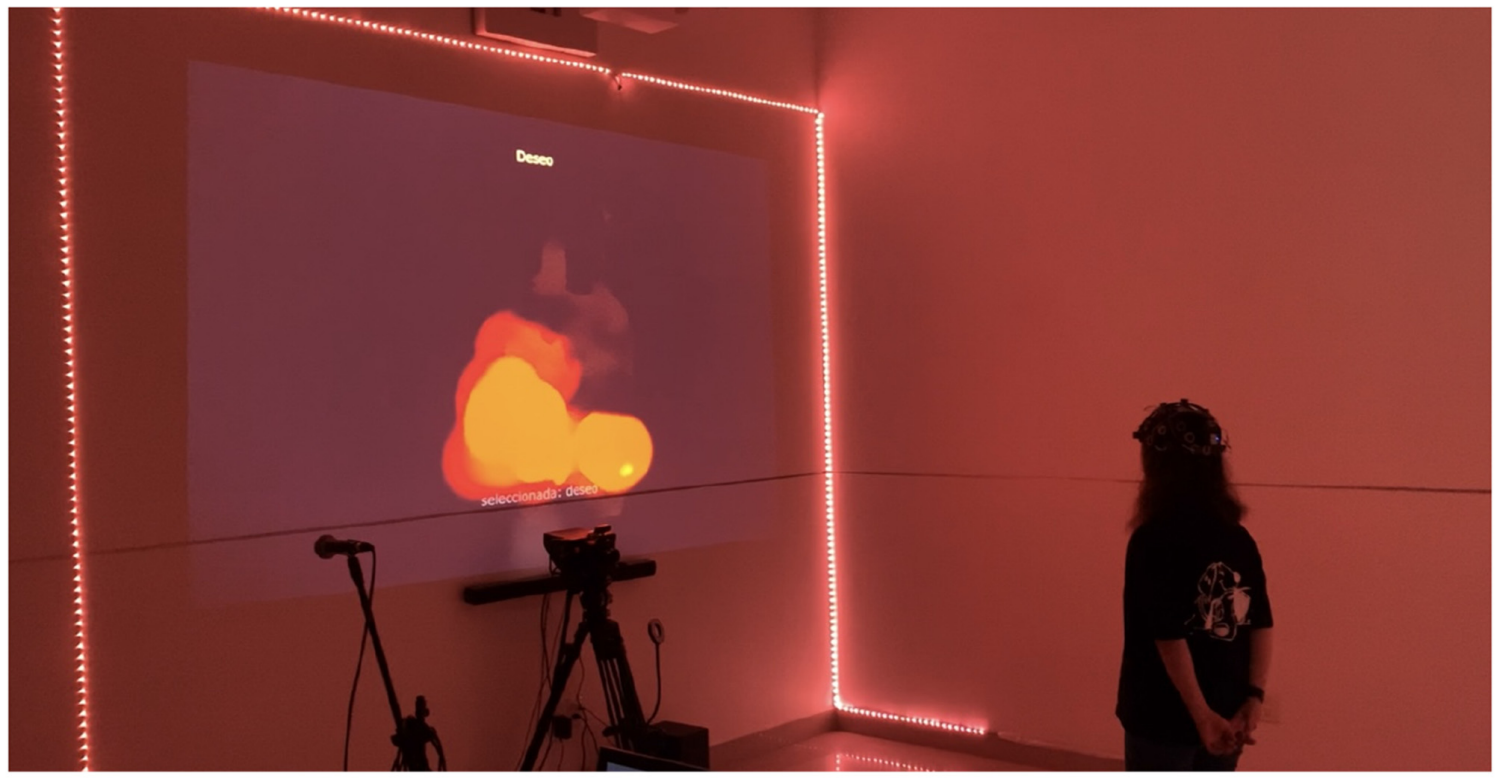
Picture of a participant of the experimental group interacting with NHLab immersive environment representing “Desire” during the first scene.

**First scene**. In the first scene (experimental group), participants were asked to portray their chosen emotion through corporal expression, which could include moving their arms and legs or walking within the NHLab experimental space. Participants were able to see their movements represented on a screen as digital brushstrokes of different colors. Using the physiological data collected, the system predicted the participant's emotional state in real time and accordingly changed the color of the brush (Blanco-Rios et al., 2024). The detected emotion also triggered changes in various aspects of the environment, including lighting color, sound, and visual elements projected on the screen. An image of the described environment is shown in Figure 2.

**Figure 3:**
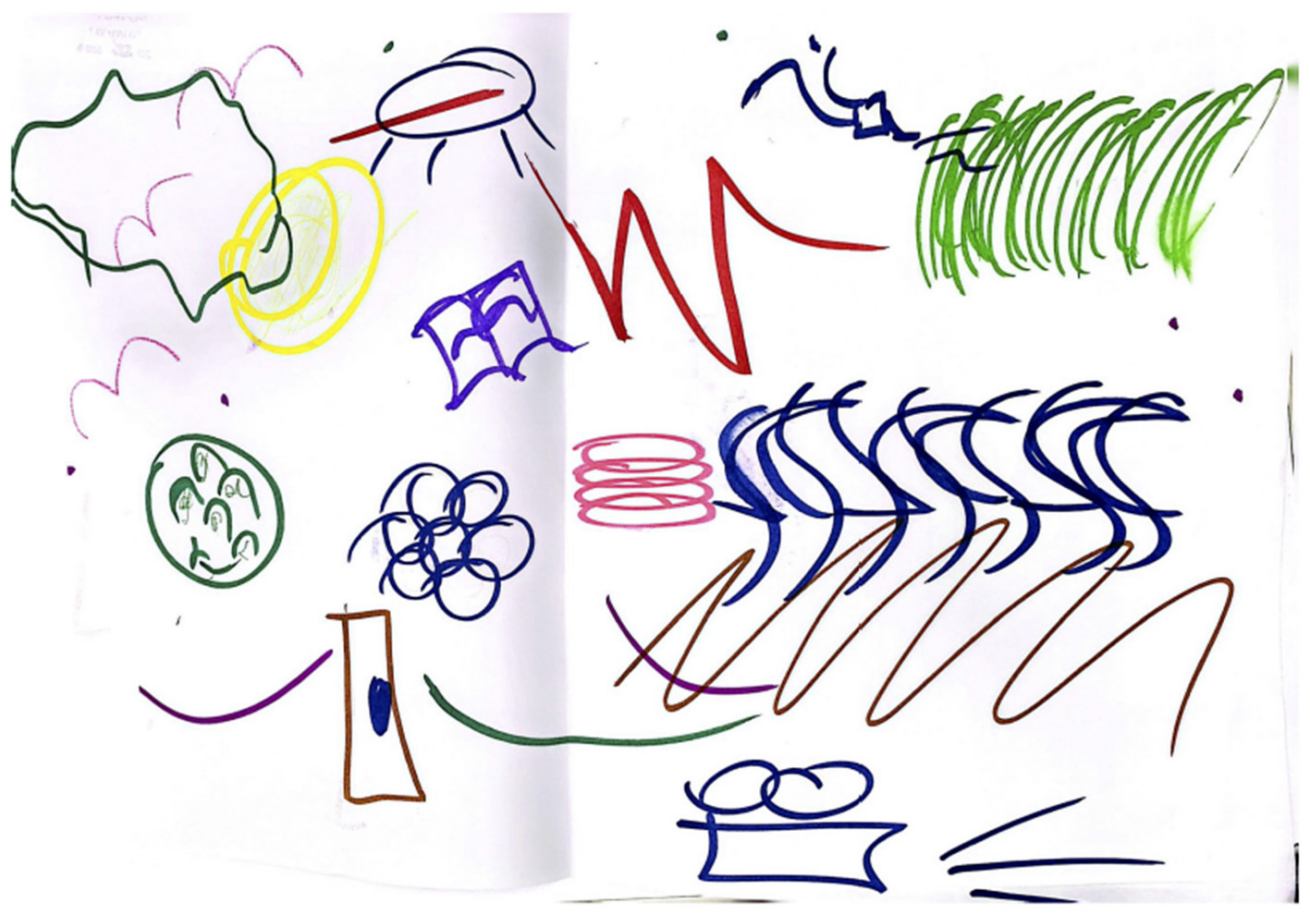
Abstract drawing representing “Joy” made by a control group participant during the first scene.

In the control group, participants were asked to portray the selected emotion through an abstract drawing. They were provided with paper, colored markers, and colored pencils, and were instructed to draw the chosen passion as they perceived and experienced it. Figure 3 presents an example of an abstract drawing created by one participant to represent “joy.” In contrast to the experimental group, this group did not receive any feedback from the system, as the environment was configured to resemble a standard classroom. Nevertheless, data from the prediction model were recorded and stored across all scenes.

**Figure 4:**
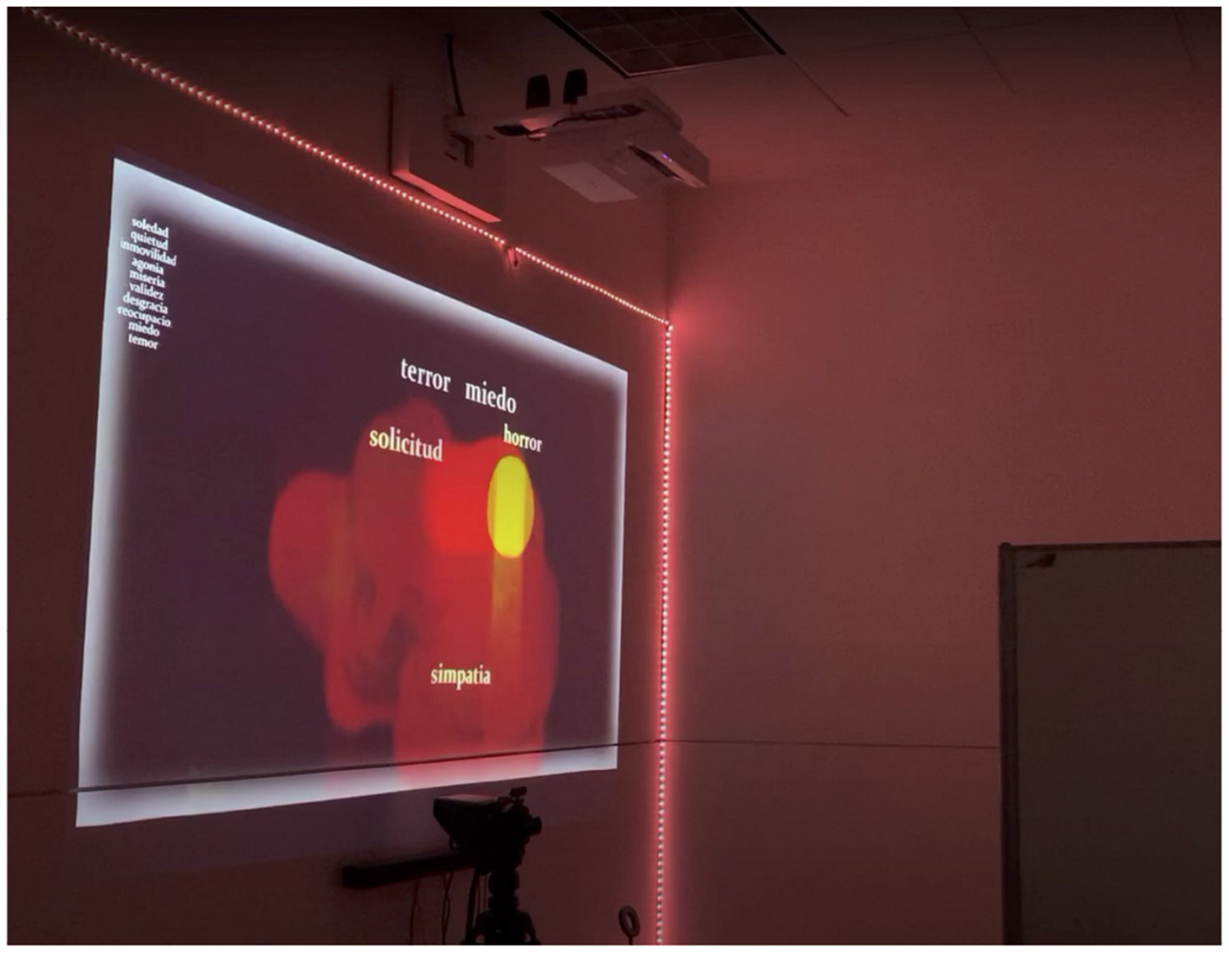
Word selection screen from the passion “sadness”, displayed to a participant during the second scene of the experimental group. Words in the screen read “terror”, “fear”, “horror”, “request”, and “sympathy”.

**Second scene**. In the second scene (experimental group), a word cloud associated with the selected passion was projected within the NHLab environment, as shown in Figure 4. Participants were asked to verbally state, using a microphone, the words that best represented their chosen passion. They were instructed to select words from the displayed word cloud or to propose new words, which were subsequently detected by the system and incorporated into the cloud.

**Figure 5:**
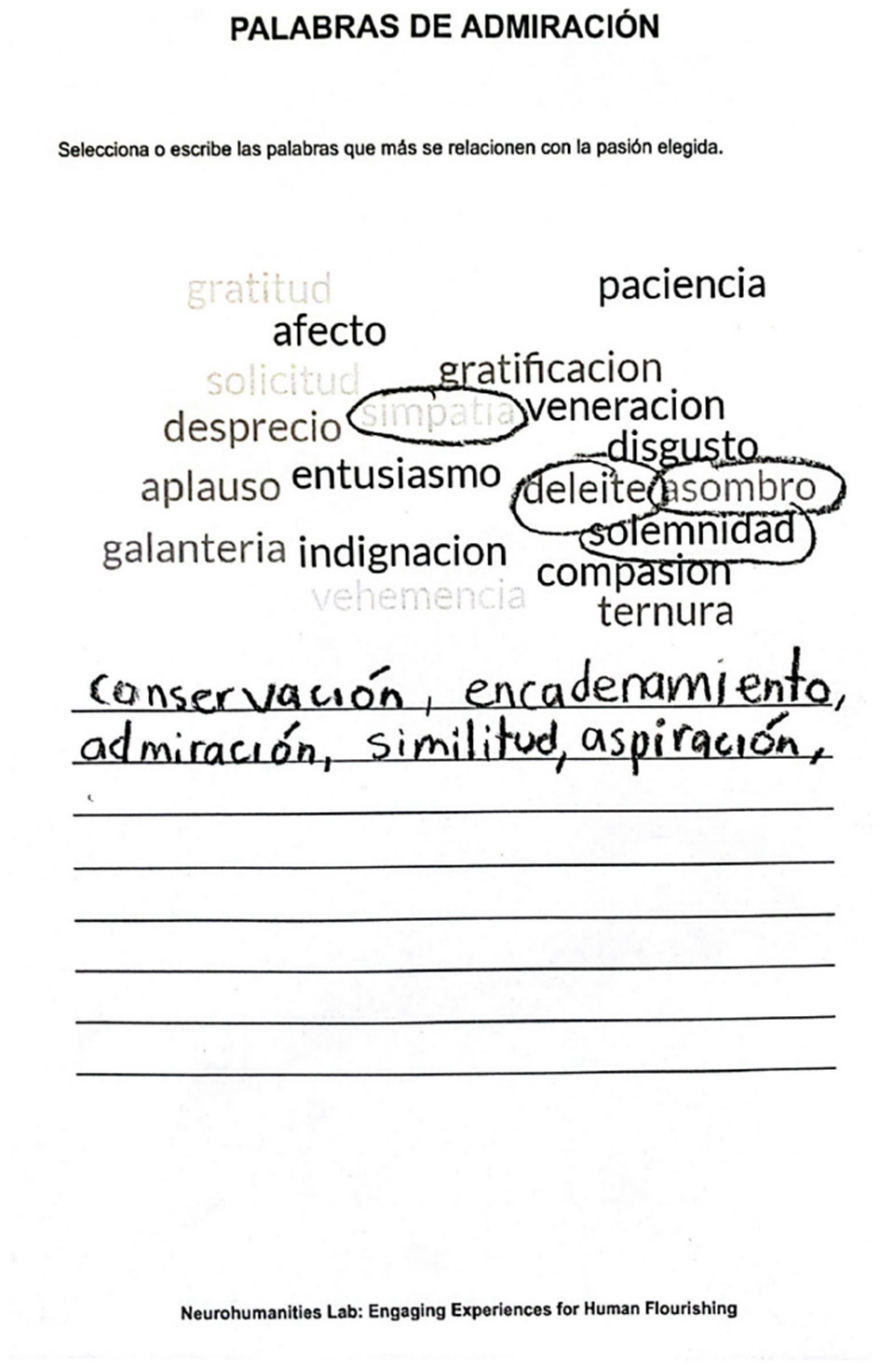
Word Selection associated with “Admiration” made by a participant in the control group during the second scene. The words encircled were “sympathy”, “delight”, “amazement” and “solemnity”, and the ones written down were “conservation”, “chaining”, “admiration”, “similarity” and “aspiration”.

For the control group, participants were provided with a printed word cloud associated with their chosen passion and were asked to encircle or write below the words they associated with the emotion as experienced during the first scene. Alternatively, they were instructed to add any missing words they felt better represented that emotion. It should be noted that these sheets were identical for all participants for each emotion and did not vary across individuals, unlike in the experimental group. An example of the activity sheet is shown in Figure 5.

**Figure 6:**
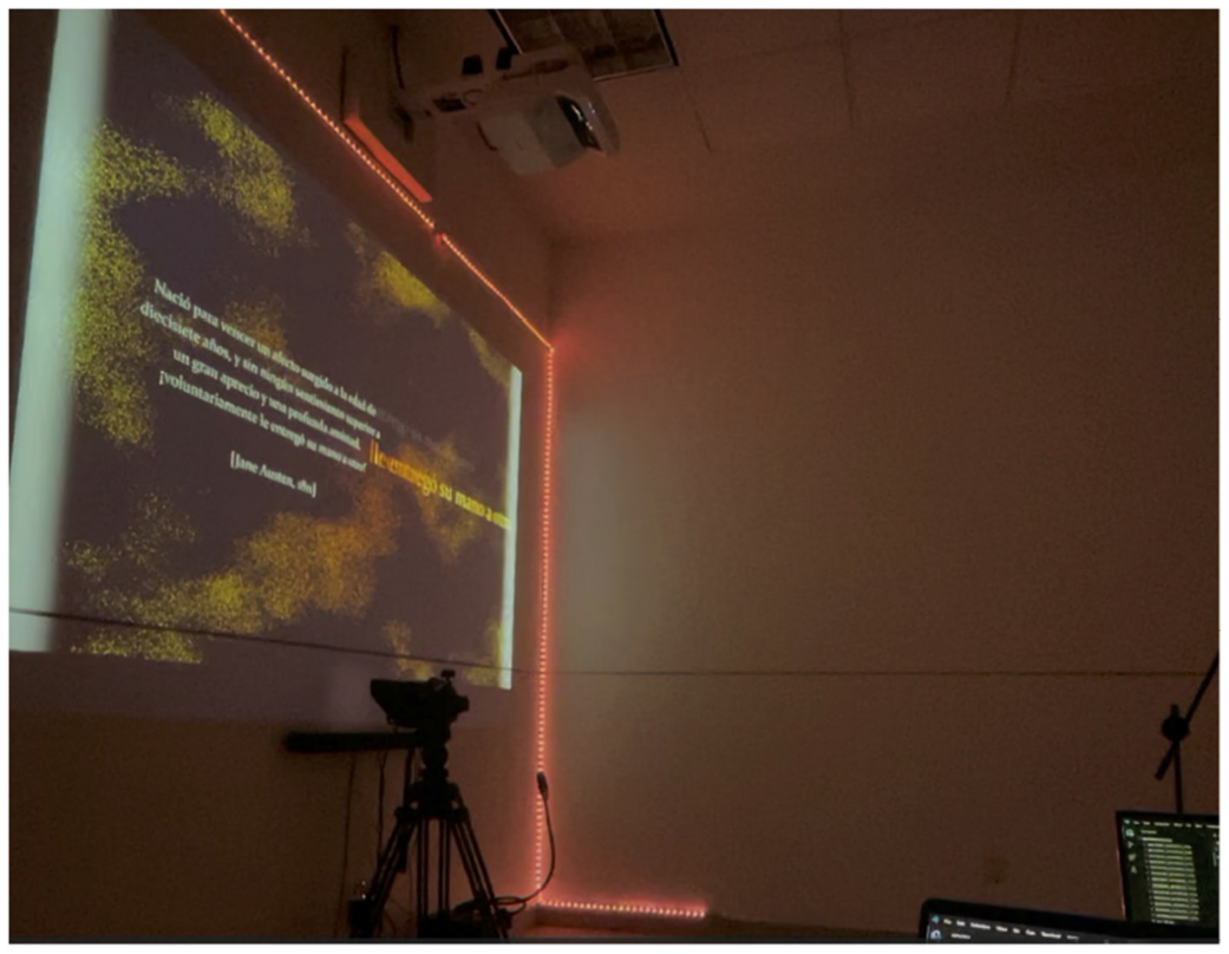
Literary fragment reading from a participant in the experimental group during the third scene.

**Third scene**. In the third scene (experimental group), literary fragments were projected onto the screen. These short fragments, consisting of one or two sentences, were related to the words selected by the participant during the second scene. Participants were asked to read the fragments aloud into a microphone in the most emphatic and comprehensible manner possible. Figure 6 illustrates how the NHLab environment changed to display the literary fragments on the screen.

**Figure 7:**
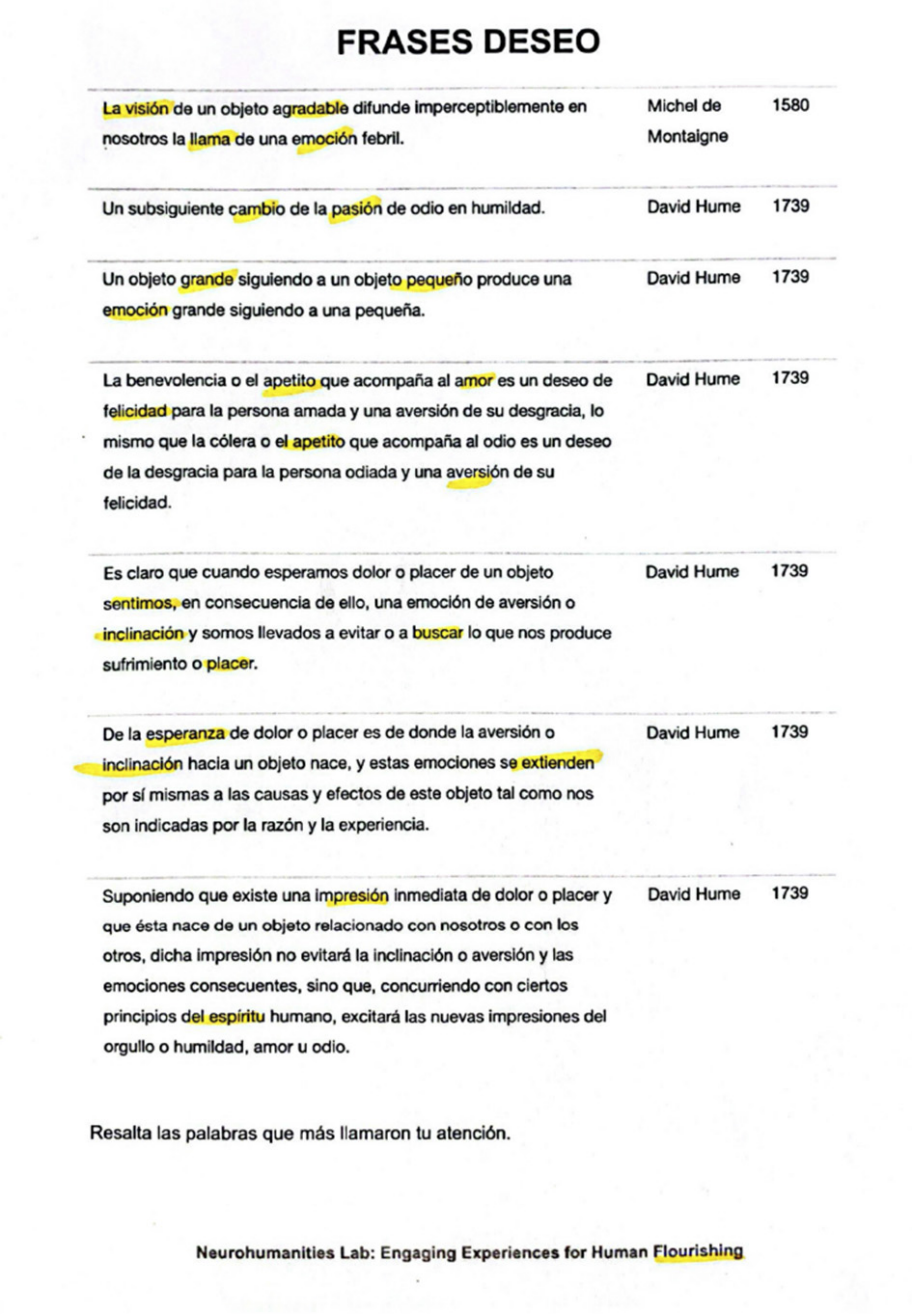
Literary fragment reading with the passion “Desire” from a participant in the control group during the third scene. Fragments included from author David Hume and Michel de Montaige. Some of the highlighted words were “the vision”, “pleasant”, “change”, “appetite”, “aversion”, “hope” and “inclination”.

For the control group, participants were provided with printed literary fragments, as shown in Figure 7. They were asked to read the fragments aloud and highlight the words that captured their attention. As in the previous scene, these sheets were identical for each emotion and did not vary across participants.

**Figure 8:**
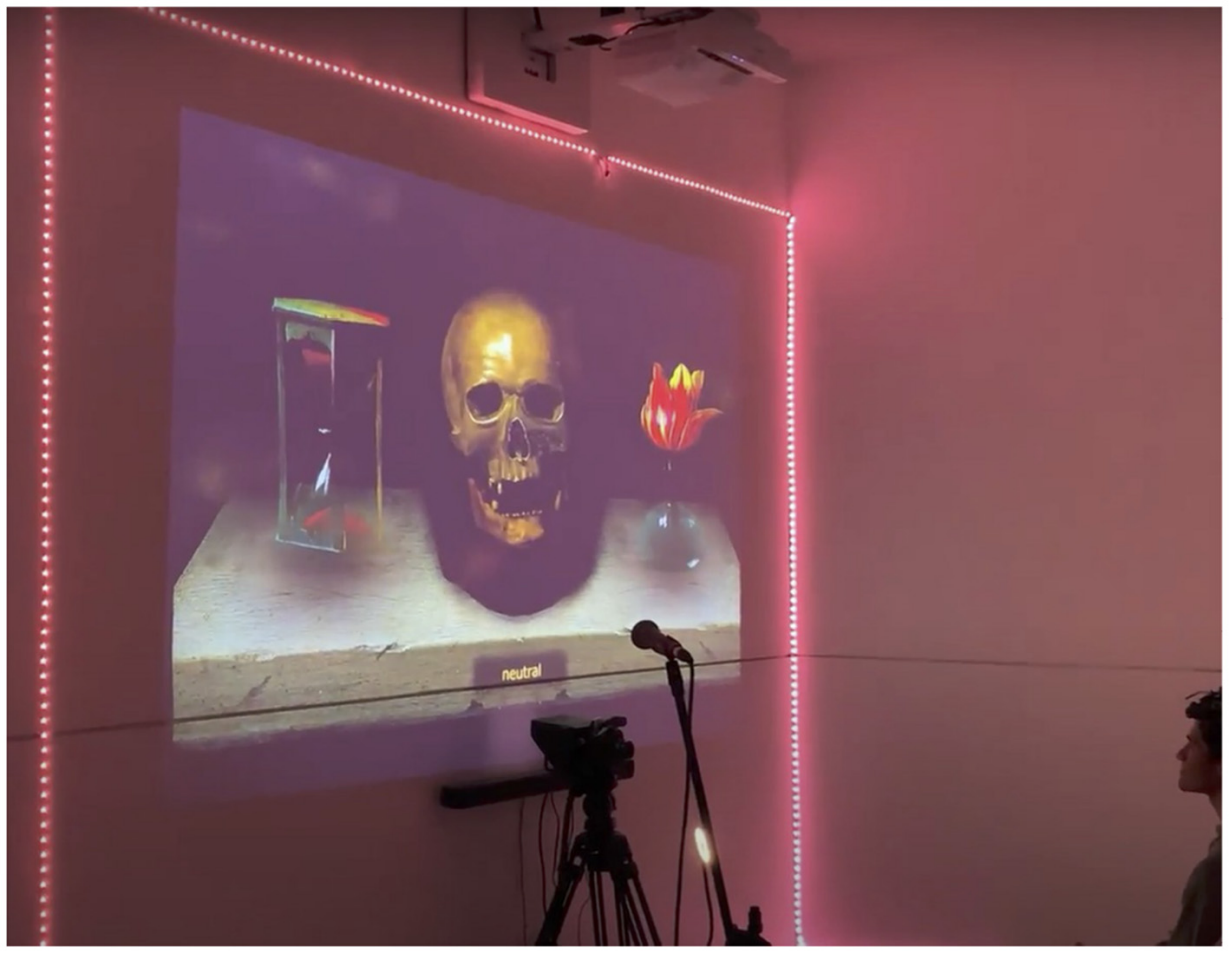
Participant in the experimental group reacting facially to the digital painting, during the fourth scene.

**Fourth scene**. During the final scene, participants in the experimental group were seated while a camera captured and recognized their facial features. A digital Vanitas painting was projected onto the screen, and participants were asked to observe it and explore the emotions it elicited through facial interaction. The sand clock and the flower within the painting changed in response to the detected facial expressions, as illustrated in Figure 8. Moreover, the skull in the center was designed to replicate the participants' head, eye, and mouth movements.

**Figure 9:**
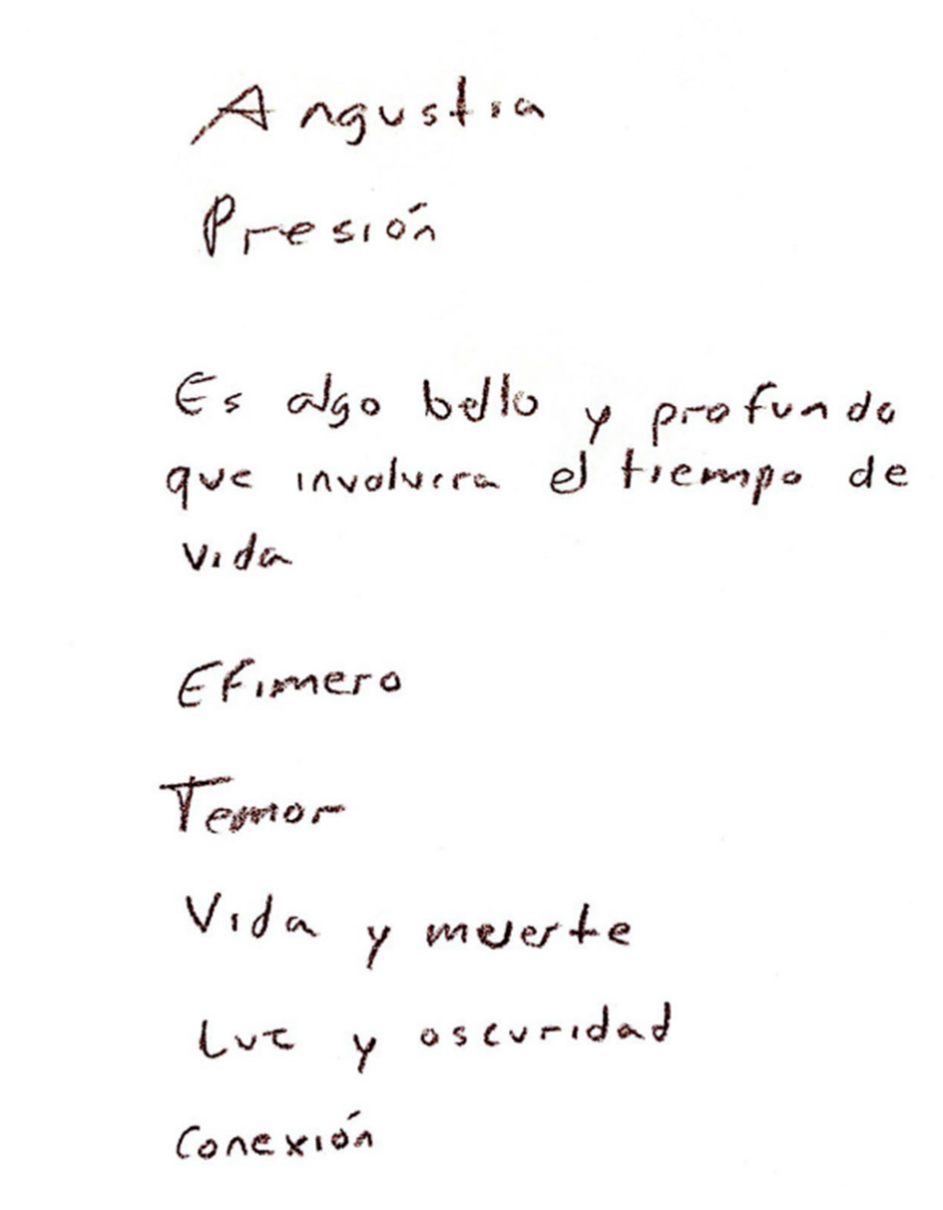
Descriptions from a painting from a participant in the control group. These translate to “anguish”, “pressure”, “it's something beautiful and deep that involves life's time”, “ephemeral”, “fear”, “life and death”, “light and darkness” and “connection”.

In contrast, participants in the control group were presented with a printed version of the painting and were asked to write a description of the feelings they experienced while observing it. Figure 9 shows an example of a description written by one participant in the control group. The same painting was used for all participants in both groups.

The purposes of the scenes follow a constructivist Kantian framework of cognitive functions, ranging from physical processes (intuition), to conceptual processes (understanding), discursive processes (reason), and judgment (imagination combined with reason) (Danielyan, 2023). Within this framework, each scene incorporates specific measurements that complement the biometric analysis conducted. These include observing the quantity and variety of movements proposed in the first scene; analyzing the emotional charge, number, and diversity of the words suggested in the second scene; examining tone of voice, as well as variations in speed and intensity, during the readings in the third scene; and assessing the diversity of emotions elicited–both from EEG recordings and facial emotion recognition–and comparing these sequences in the fourth scene.

### 2.3 Data analysis

#### 2.3.1 Data pre-processing

The EEG signals acquired from the OpenBCI cap, along with the EDA and BVP signals recorded using the Empatica E4 wristband, were used for further analysis and comparison between groups.

EEG signals were collected using custom Python scripts at a sampling rate of 250 Hz. Both earlobes were used as reference points, and data were recorded from eight channels–Fp1, Fp2, C3, C4, P7, P8, O1, and O2–following the standard 10–20 International System. The data were preprocessed as described in Romo-De León et al. (2024). During preprocessing, the MATLAB PREP Pipeline plug-in was employed, which detrends the signal, removes line noise without relying on a fixed filtering method, and re-references the signal relative to an estimate of the “true” average reference. Specifically, line noise at 60 Hz was removed using a CleanLine approach with a scan bandwidth of ± 2 Hz around the target frequency and a taper window size of 4 seconds with a 1-second step. All channels were included in both the referencing and noise removal procedures (Bigdely-Shamlo et al., 2015; The MathWorks, n.d.b).

**Figure 10:**
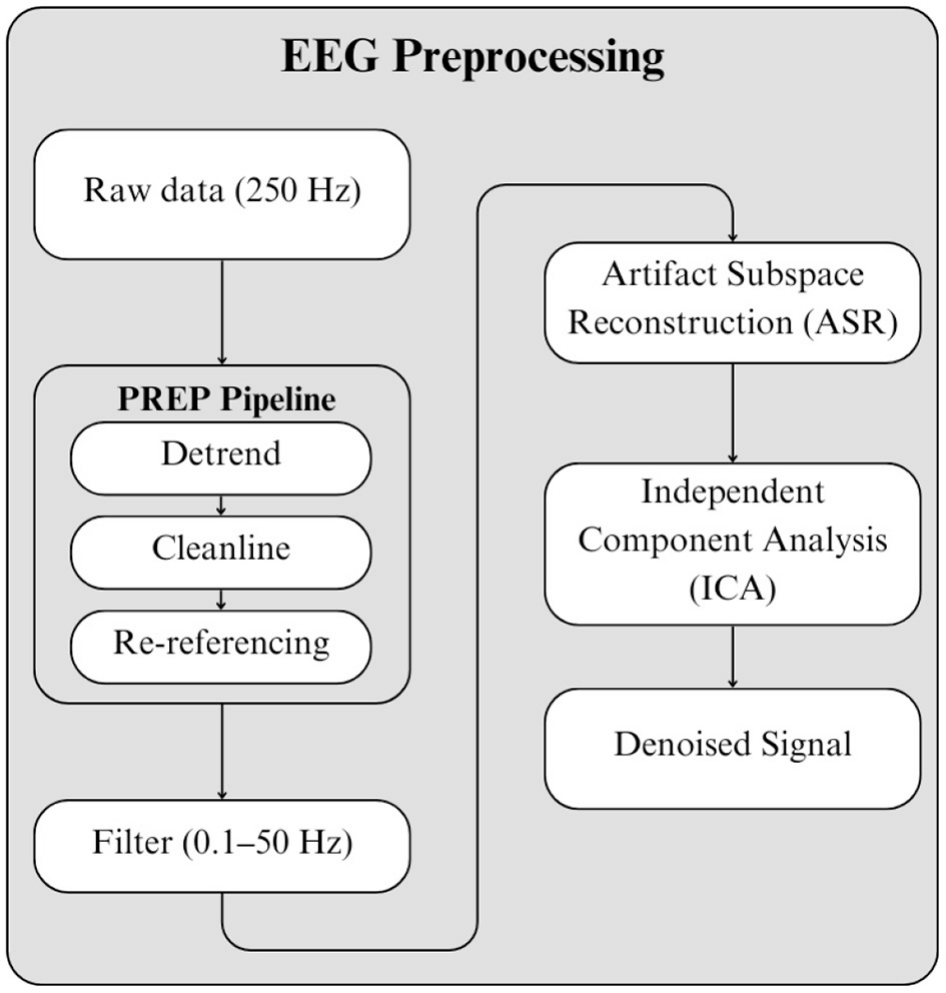
EEG pre-processing steps (Romo-De León et al., 2024).

Following this step, a 4th order, Butterworth 0.1–50 Hz bandpass filter was applied to remove baseline drifts while preserving relevant neural frequency components. Subsequently, two artifact removal methods were applied: Artifact Subspace Reconstruction (ASR) and Independent Component Analysis (ICA). ASR was performed using a burst detection threshold of 15 standard deviations, while flatline, correlation, and line noise thresholds were disabled. Bad channels were iteratively interpolated until no channels were classified as bad (Chang et al., 2018). ICA was then conducted using the runica algorithm. Due to the use of eight EEG channels, Principal Component Analysis (PCA) was applied to reduce the data to eight dimensions prior to ICA, ensuring compatibility with the dataset size (Debener et al., 2010). Components exhibiting artifact-like spatial, temporal, and spectral characteristics, and identified as artifacts by the ICLABEL plug-in, were removed. The overall preprocessing procedure is illustrated in Figure 10.

For EDA data analysis, the cvxEDA MATLAB application was used (Greco et al., 2016a). This application decomposes the signal into tonic and phasic components, as well as an additive noise term, using a convex optimization approach (Greco et al., 2016a). As this method explicitly accounts for noise and artifacts, no additional preprocessing steps were applied. The BVP signal was also used for further analysis and was analyzed in its raw form.

#### 2.3.2 EEG data analysis

After preprocessing, the cleaned EEG signals were used for further analysis using machine learning (ML) algorithms (Alzubi et al., 2018). One of the primary research questions was to determine which differences in participants' EEG signals could be distinguished when comparing the two learning experiences: the traditional classroom set-up and the partially immersive set-up. Accordingly, feature extraction and ML techniques were employed to identify which features most effectively supported the discrimination between these two conditions (Moinnereau et al., 2022; Grassini and Laumann, 2020).

Feature matrices were built to evaluate classification accuracy of multiple ML models. Each feature matrix comprised EEG frequency-domain features, including total power and power spectral density (PSD) across all frequency bands—delta (1–4 Hz), theta (4–8 Hz), alpha (8–12 Hz), beta (12–30 Hz), and gamma (30–100 Hz)—for all eight electrodes, as well as the engagement index ratio derived from the frontal electrodes (Fp1 and Fp2), as shown in Equation 1.


$$\begin{array}{l} Engagement\;Index=\frac{Beta}{Alpha+Theta} \end{array}$$ (1)

These calculations were performed on a per-second basis for each recording, participant, and scene. Several review studies have reported the use of frequency-domain EEG analysis to distinguish and characterize immersive and non-immersive technological experiences (Moinnereau et al., 2022; Grassini and Laumann, 2020). Custom MATLAB code was developed to compute the features and construct a feature matrix (160 × 83; seconds per scene × features and target vector) containing the estimated values. Band power was calculated using MATLAB's bandpower function (The MathWorks, n.d.a), and power spectral density (PSD) was estimated using Welch's method via the pwelch function (The MathWorks, n.d.d). Both band power and PSD have been associated with presence and engagement, as they may increase or decrease depending on the level of immersion (Moinnereau et al., 2022; Grassini and Laumann, 2020).

**Figure 11:**
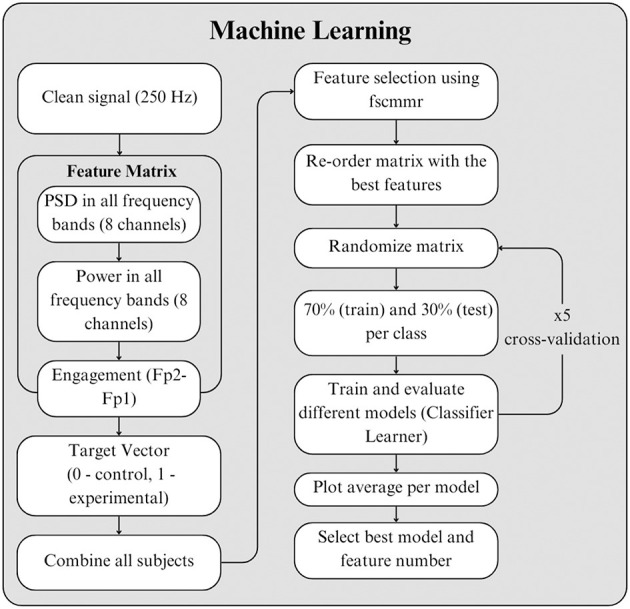
Steps to identify best features and best ML algorithm model.

The minimum redundancy maximum relevance (MRMR) algorithm was applied to identify the most informative predictors from the feature matrices (The MathWorks, n.d.c). A separate feature matrix was constructed for each scene to enable comparison of EEG features between the two groups. For each feature matrix, data from 70% of the participants were used for training, while data from the remaining 30% were used for testing. Three ML algorithms were evaluated: decision tree, linear discriminant analysis, and quadratic discriminant analysis. Five-fold cross-validation was performed across all three models to prevent overestimation, with participants randomly selected at each iteration. Figure 11 presents a visual flowchart of the procedure used to identify the best-performing ML model.

Pre-processed EEG signals were analyzed using dipole fitting to identify dipole representations of brain sources based on the ICA method (EEGLAB, n.d.b). Subsequently, neighboring dipoles were grouped into clusters; the resulting centroids were then located and classified within specific Brodmann Areas (Guy-Evans, n.d.). This analysis was performed using the DIPFIT plugin for MATLAB (EEGLAB, n.d.a).

**Figure 12:**
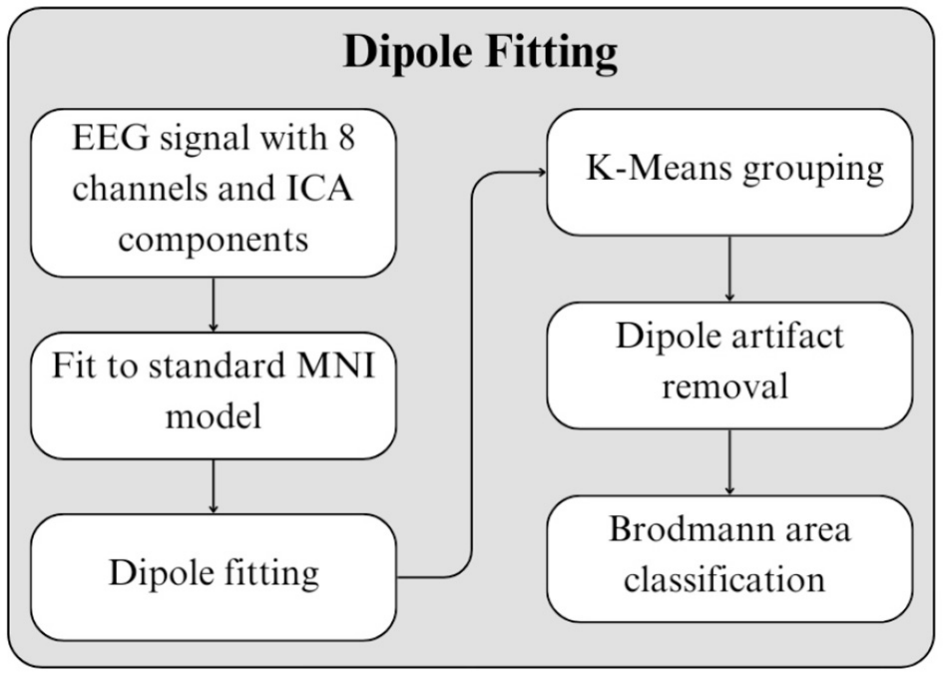
Steps to identify dipoles from ICA, and classify them into Brodmann Areas.

To determine the optimal number of clusters, we applied an optimization approach suggesting between 5 and 15 clusters based on three specific algorithms (Calinski-Harabasz, Silhouette). Dipoles were grouped by experimental condition, yielding distinct clusters for the Control and Experimental groups for each scene. The dipole fitting workflow is illustrated in Figure 12.

#### 2.3.3 EDA data analysis

The cvxEDA model was applied to the raw EDA signals to extract the phasic component, utilizing default parameters for all subjects (Greco et al., 2016a). From this component, the SCR count and phasic amplitude were extracted as primary features. In accordance with established protocols, a minimum threshold of 0.05 μ*S* was set to identify significant SCRs (Posada-Quintero and Chon, 2020; Greco et al., 2016b). This procedure was applied to all subjects across all scenes, including the baseline. Following extraction, the mean SCR count and amplitude were calculated for both the Control and Experimental groups.

#### 2.3.4 BVP data analysis

Heart rate (HR) was derived from the BVP signals for each subject. Using MATLAB's *findpeaks* command, we identified the signal's local maxima. To prevent heart rate calculation errors caused by the dicrotic notch, only the secondary peak was considered for each identified maximum (Peper et al., 2007). The time intervals between successive peaks were calculated and converted to an estimated heart rate by dividing by 60 s.

## 3 Results and discussion

### 3.1 EEG analysis

#### 3.1.1 Machine learning

The MRMR algorithm was employed to rank the feature matrices and identify those that most effectively distinguished between the control and experimental classes. Table 1 presents the top 10 features identified for each scene. Across all scenes, the engagement level of one or both electrodes consistently emerged as a key differentiator. This aligns with existing literature suggesting that engagement is a critical factor for quantifying immersion and correlates strongly with the level of presence (Bond et al., 2020; Debener et al., 2010). Furthermore, because the engagement feature was ranked as highly relevant by the algorithm, it may serve as a potential neuromarker for evaluating the knowledge or understanding acquired by each group. High levels of engagement may indirectly indicate superior learning outcomes (Ochs and Sonderegger, 2022).

**Table 1:** 10 best features per scene after being sorted by the MRMR algorithm.

**Scene**	**10 best features**	
Scene 1	FP2_beta	8_theta_psd
	fp1_engagement	P7_delta
	O1_delta	FP1_gamma_psd
	C4_delta_psd	C4_alpha
	FP2_gamma	O2_gamma_psd
Scene 2	C3_alpha	FP1_gamma_psd
	O2_delta_psd	P7_theta
	O2_gamma	P8_beta_psd
	fp2_engagement	O2_theta
	C3_delta	C4_delta_psd
Scene 3	FP2_beta	FP1_alpha
	C3_gamma_psd	FP2_theta
	fp1_engagement	P8_theta_psd
	P8_delta_psd	O1_delta
	O1_alpha_psd	FP2_alpha
Scene 4	P8_delta	P8_beta
	fp2_engagement	P8_theta_psd
	FP1_gamma	FP2_alpha_psd
	O1_theta_psd	O2_beta
	P7_alpha	C4_gamma

**Figure 13:**
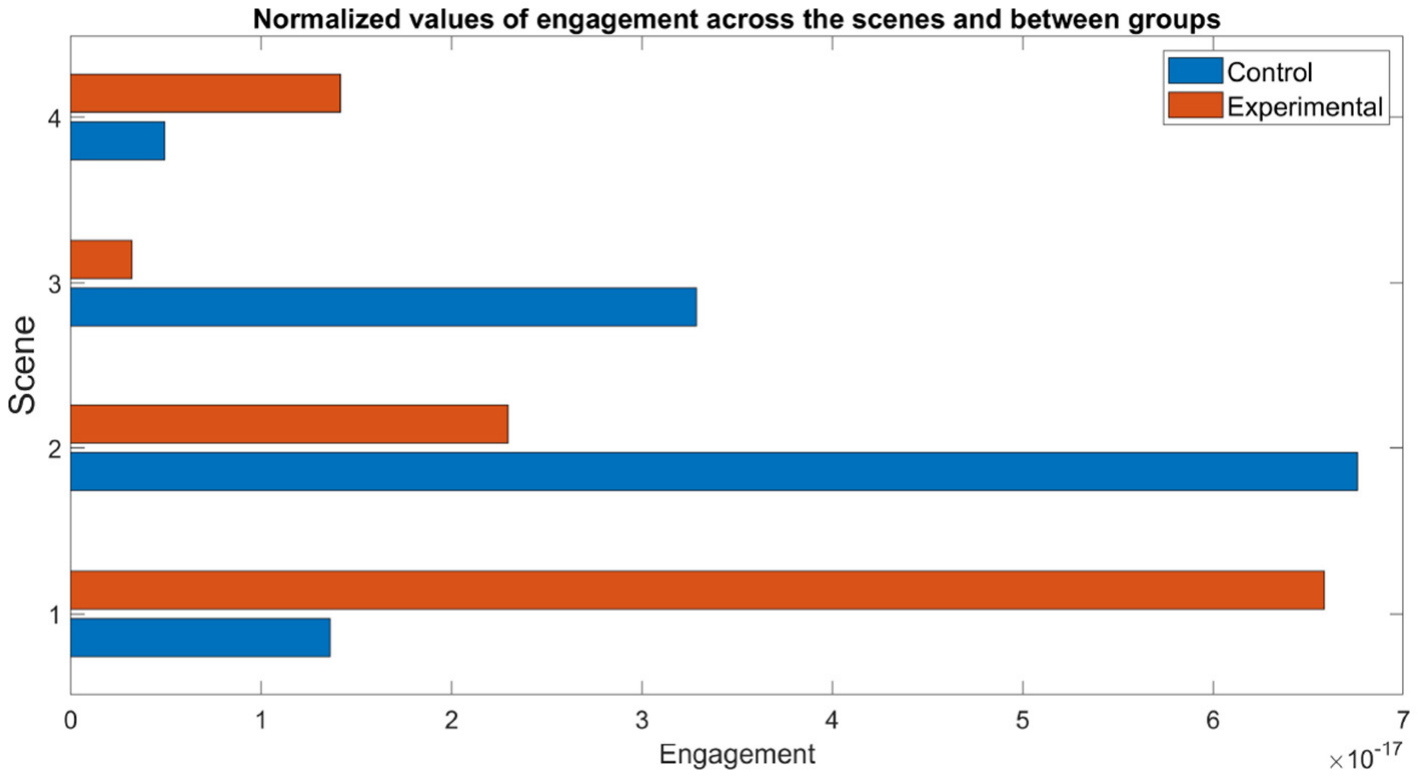
Engagement index estimations across all four scenes, for control and immersive groups.

The engagement index values were averaged across scenes and groups, and are are illustrated in Figure 13. In Scene 1, engagement values exhibited a sharp contrast, with the experimental group demonstrating significantly higher engagement. Conversely, during Scenes 2 and 3, the control group showed higher engagement levels, suggesting that these specific activities may have been more engaging within a traditional classroom setting. Finally, in Scene 4, the data again revealed higher engagement within the experimental group.

**Figure 14:**
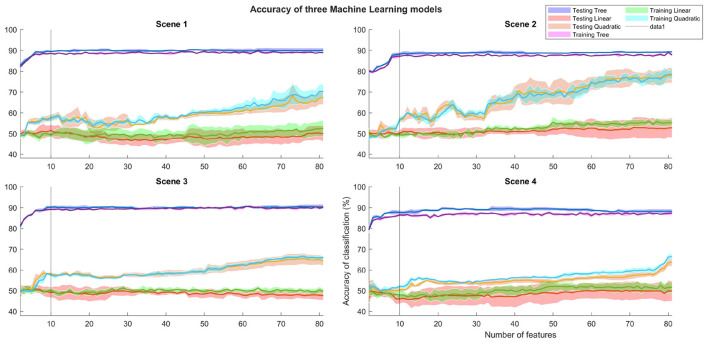
Machine Learning Models performance across all four scenes. Model accuracy was evaluated considering an increasing amount of features, using five cross-validations per model. Thick line and shaded area represent mean and standard deviation per model accuracy respectively.

Machine learning (ML) was employed to develop models capable of identifying the most relevant biometric patterns that distinguish between the control and immersive groups. The accuracy levels for each scene were evaluated using three supervised methods: Decision Tree, Linear Discriminant Analysis, and Quadratic Discriminant Analysis. A comparison of these models across scenes is illustrated in Figure 14. The y-axis depicts the average accuracy of each model (across cross-validations) relative to the increasing number of features on the x-axis.

Accuracy is reported for both testing and training datasets; colored, thick lines represent the mean accuracy, while the shaded areas indicate the standard deviation across cross-validations. From the 10-feature threshold onward, the Decision Tree model demonstrates the highest performance, reaching nearly 90% accuracy and maintaining consistent results as the number of features increases.

In Table 2, the maximum accuracy achieved and the corresponding number of features can be observed for each scene and each Machine Learning model.

**Table 2:** Number of features with the highest average accuracy for the Machine Learning models.

**Scene**	**Decision tree**	**Linear discriminant**	**Quadratic discriminant**
1	76 features (94.77%)	9 features (57.07%)	64 features (81.93%)
2	80 features (92.79%)	8 features (52.60%)	79 features (82.93%)
3	81 features (93.80%)	34 features (55.58% )	80 features (69.23% )
4	65 features (93.48%)	32 features (56.86%)	82 features (63.61%)

Table 3 presents the average accuracy for each model using a 10-feature set. This threshold was selected because the Decision Tree model's average accuracy stabilizes at approximately 90% beyond this point. In contrast, the Linear and Quadratic Discriminant Analysis methods show consistently lower accuracy values, even when utilizing the same 10 features.

**Table 3:** Average accuracy percentage of Machine Learning models with 10 features.

**Scene**	**Decision tree**	**Linear discrimination**	**Quadratic discrimination**
1	92.80%	56.03%	62.57%
2	91.82%	49.15%	52.05%
3	92.27%	53.01%	54.69%
4	92.44%	55.33%	56.95%

#### 3.1.2 Dipole fitting

Dipole fitting enables the 3D localization of independent components (brain sources) within specific regions of the cerebral cortex. In this analysis, subjects were categorized into control and experimental groups across four distinct scenes. This allowed for the identification of active brain sources for both the control and immersive groups throughout Scenes 1 to 4. Only independent components with DIPFIT residual variance below 20% were retained for source localization and clustering analyses.

Following the clustering of these dipoles, the resulting clusters were mapped to the Montreal Neurological Institute (MNI) template to identify their corresponding Brodmann Areas (BAs). Table 4 summarizes the BAs activated during each scene for both the control and experimental groups (Akalin Acar and Makeig, 2013).

**Table 4:** Brodmann Areas analysis.

**Scene**	**Control group**	**Both**	**Experimental group**
1	-	10, 18, 19	7
2	37, 39	10, 18	9, 40
3	6	10, 18, 19	1
4	19	6, 10, 18	-

**Figure 15:**
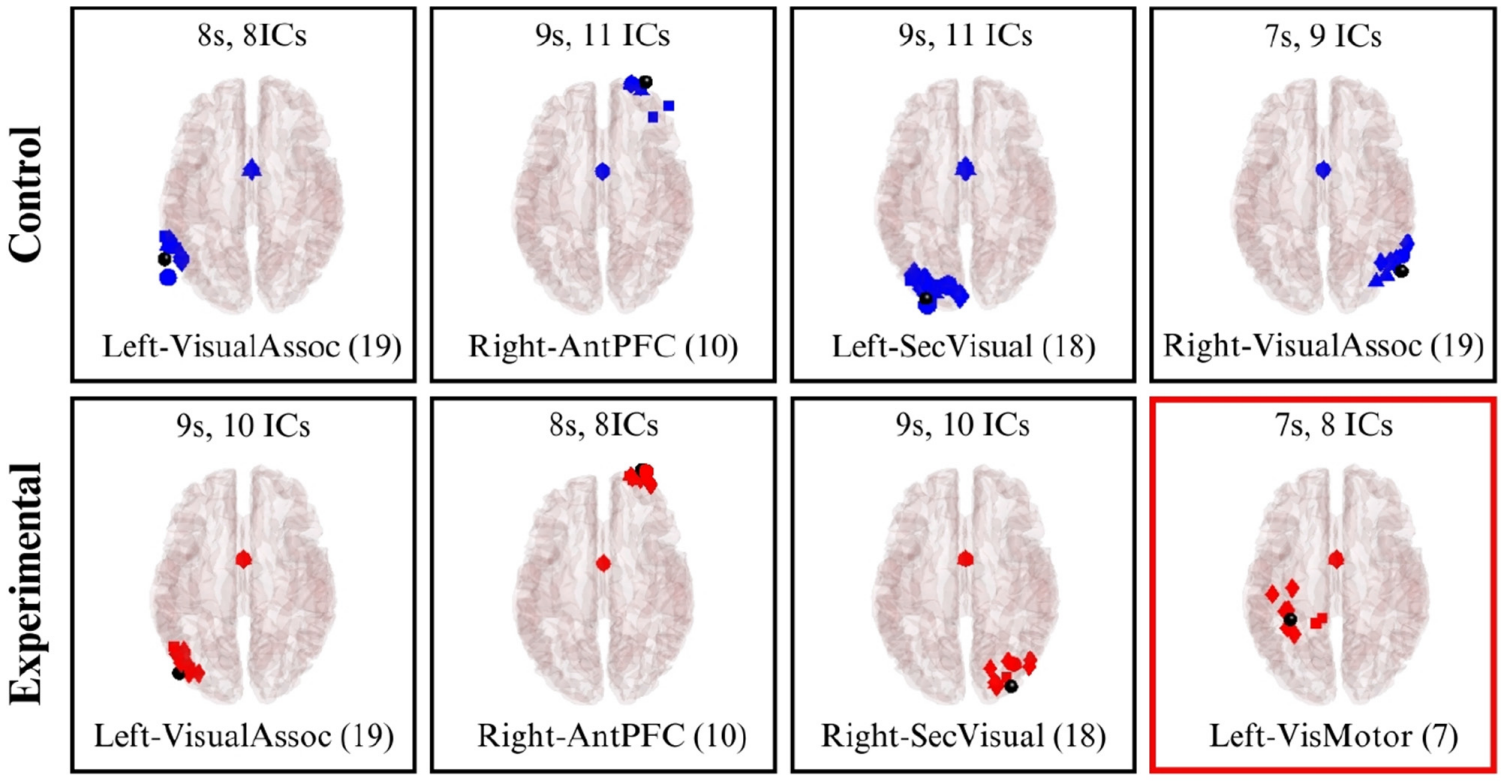
Brodmann Areas found during the dipole fitting and clustering analysis for Scene 1 (experimental and control groups). Colored boxes show unique BA activations in one particular group.

In Scene 1, three clusters were identified in both the control and experimental groups. The first cluster was located in BA 10 (Anterior prefrontal cortex), an area involved in higher cognitive functions such as planning and task management. This activity likely reflects the cognitive demands of creating an abstract painting, which requires a degree of strategic planning. Additionally, clusters were identified in BA 18 and BA 19 [Secondary Visual Cortex (V2) and Associative Visual Cortex (V3, V4 & V5)], which are associated with receiving and analyzing complex visual information. These findings directly correlate with the visual tasks performed by both groups. Notably, the experimental group also exhibited activation in BA 7 (Somatosensory Association Cortex). This activation is associated with processing sensory stimuli and may reflect the multi-sensory experience of the immersive space, as participants visualize themselves within the projection. These clusters are visualized in Figure 15.

**Figure 16:**
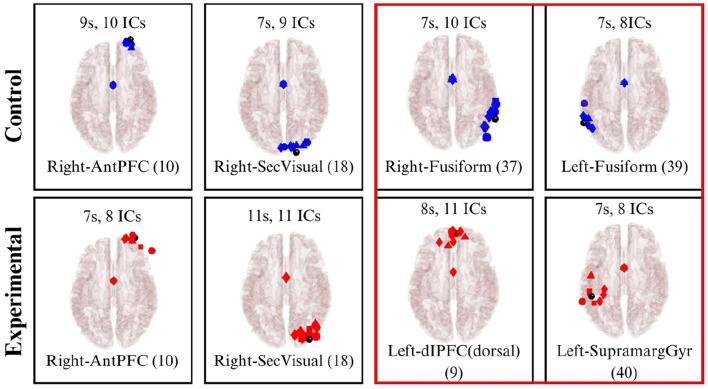
Brodmann Areas found during the dipole fitting and clustering analysis for Scene 2 (experimental and control groups). Colored boxes show unique BA activations in one particular group.

In Scene 2, observations were similar to Scene 1, with clusters identified in BAs 10 and 18 for both groups. However, the experimental group uniquely exhibited clusters in BA 9 (Dorsolateral prefrontal cortex) and BA 40 (Supramarginal gyrus). These areas are associated with executive functions—including memory and attention—as well as phonological processing and emotional responses. This activation likely reflects the cognitive demands of recalling and articulating words related to Descartes' passions. In contrast, the control group showed a cluster in BA 37 (Fusiform gyrus), which is linked to high-level visual processing such as word recognition. This may correspond to the participants' interaction with the physical word list and the associated reading comprehension tasks. The dipole clustering for Scene 2 is illustrated in Figure 16.

**Figure 17:**
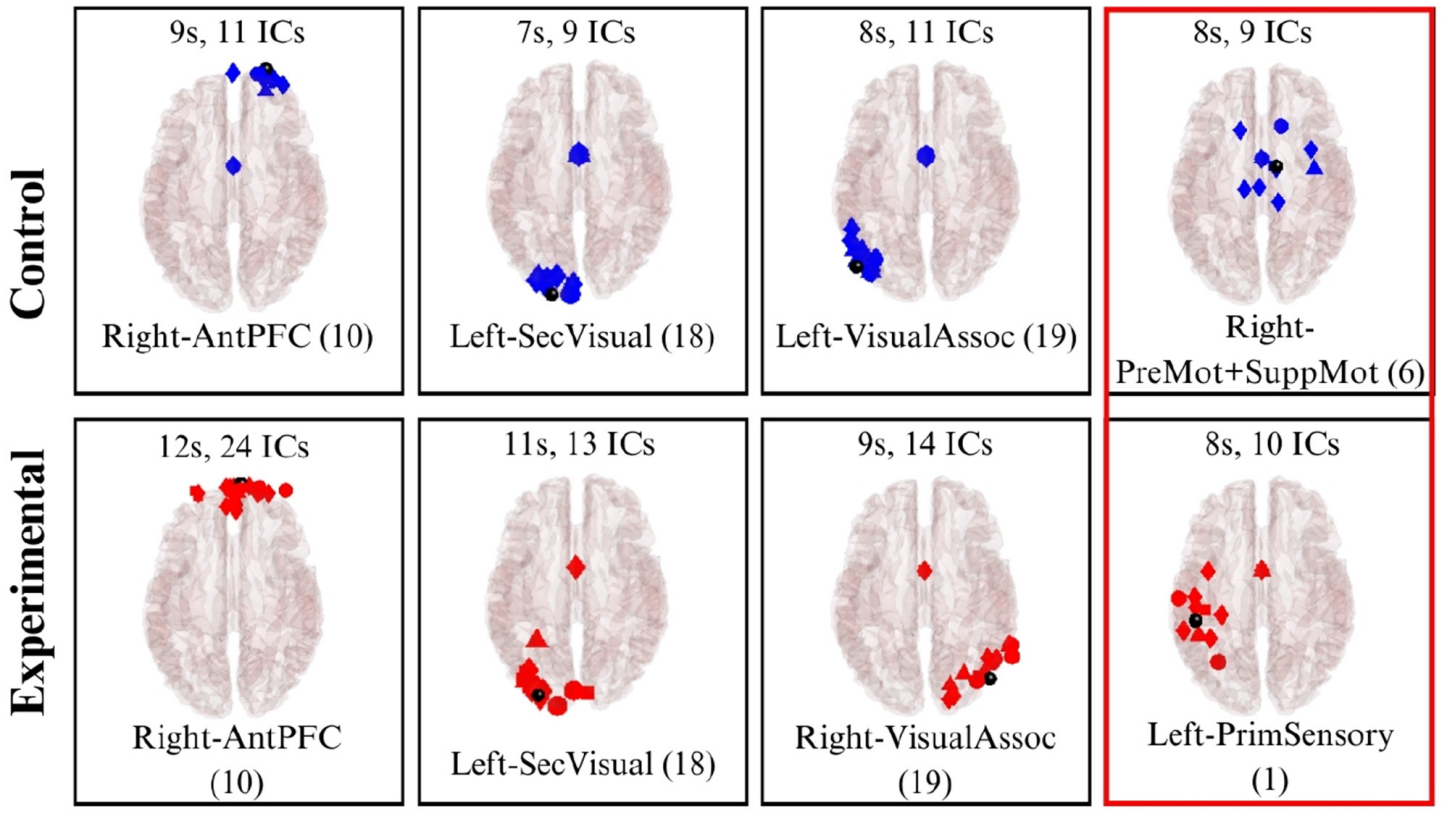
Brodmann Areas found during the dipole fitting and clustering analysis for Scene 3 (experimental and control groups). Colored boxes show unique BA activations in one particular group.

Similar to the previous scenes, Scene 3 exhibited clusters of brain activity in BAs 10, 18, and 19 for both groups (Figure 17). In the experimental group, activation was uniquely observed in BA 1 (Primary somatosensory cortex), which is responsible for processing somatic sensations. This activation may be linked to the subjects observing recordings of their own movements from Scene 1, potentially eliciting a higher level of proprioception. Conversely, the control group showed a cluster in BA 6 (Premotor and Supplementary Motor Cortex), an area associated with movement planning and control. This activation likely reflects the physical task requirements in the control condition, where participants manually selected literary quotes, whereas the experimental group read the quotes statically as they were projected.

**Figure 18:**
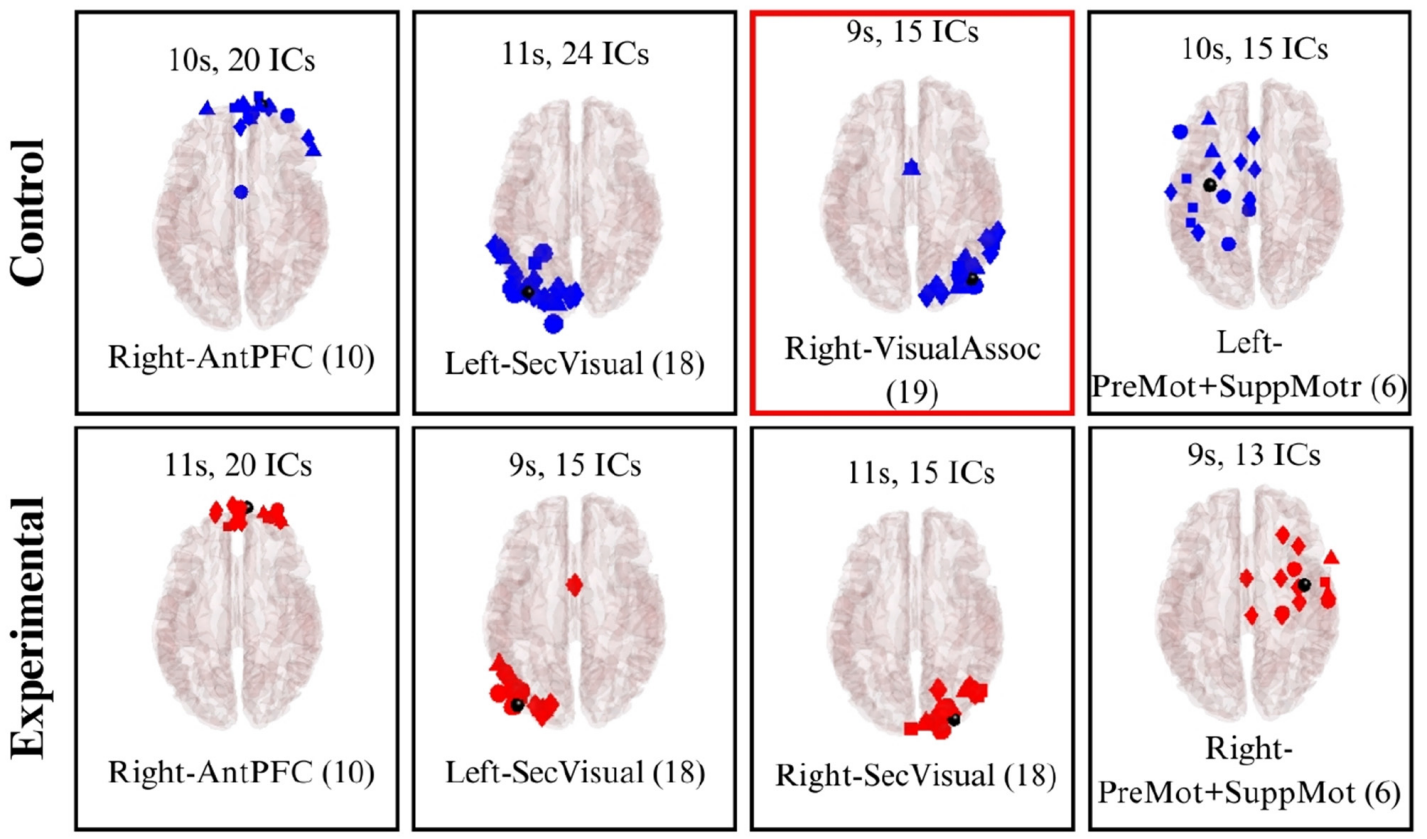
Brodmann Areas found during the dipole fitting and clustering analysis for Scene 4 (experimental and control groups). Colored boxes show unique BA activations in one particular group.

Finally, in Scene 4 (Figure 18), clusters were identified in both groups within BA 6, BA 10, and BA 18 (the premotor cortex, anterior prefrontal cortex, and secondary visual cortex, respectively). Notably, the control group uniquely exhibited a cluster in BA 19 (Associative Visual Cortex). This area is responsible for processing complex visual information and likely reflects the cognitive demands associated with interpreting the specific painting presented to the control group in this scene.

### 3.2 EDA analysis

**Figure 19:**
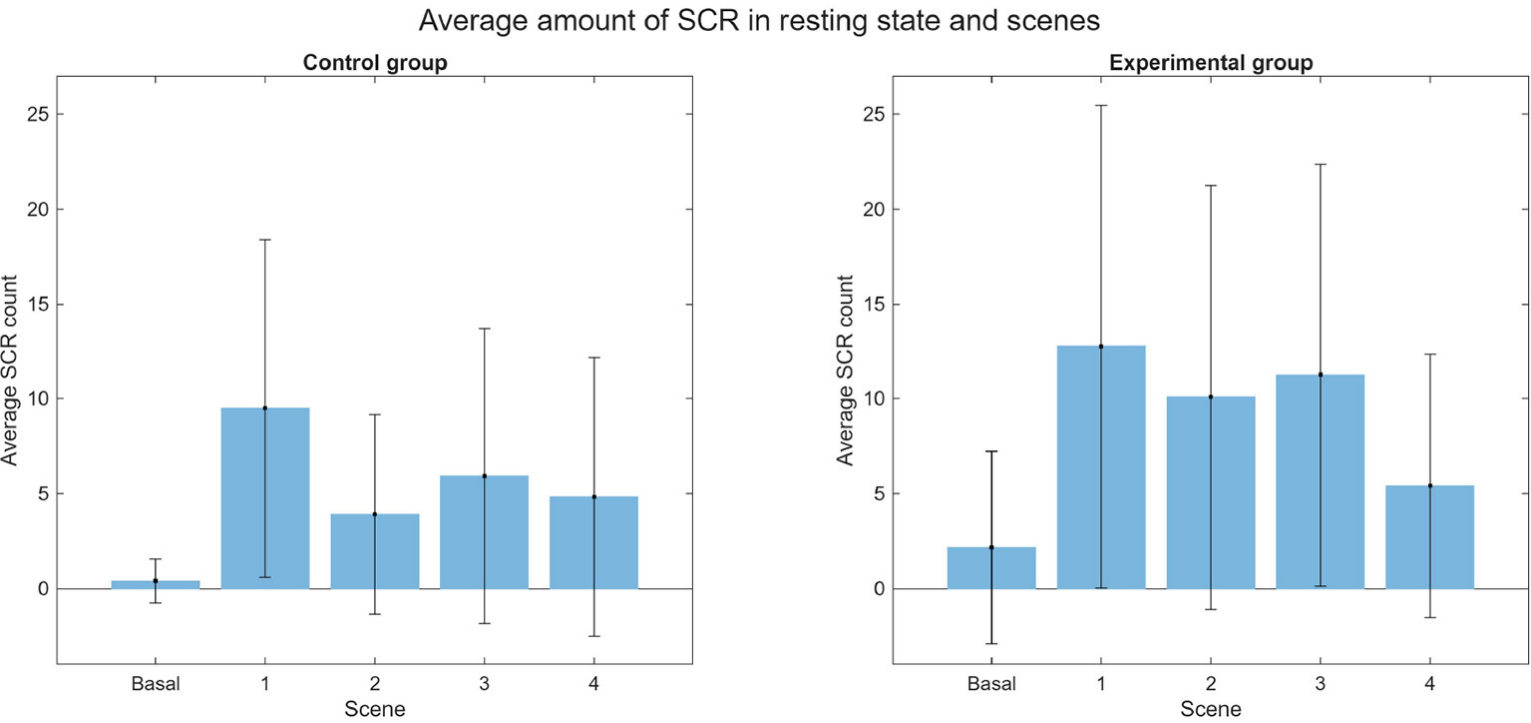
Average amount of SCR of EDA in basal state and scenes separated by control (**left**) and experimental (**right**) groups.

**Figure 20:**
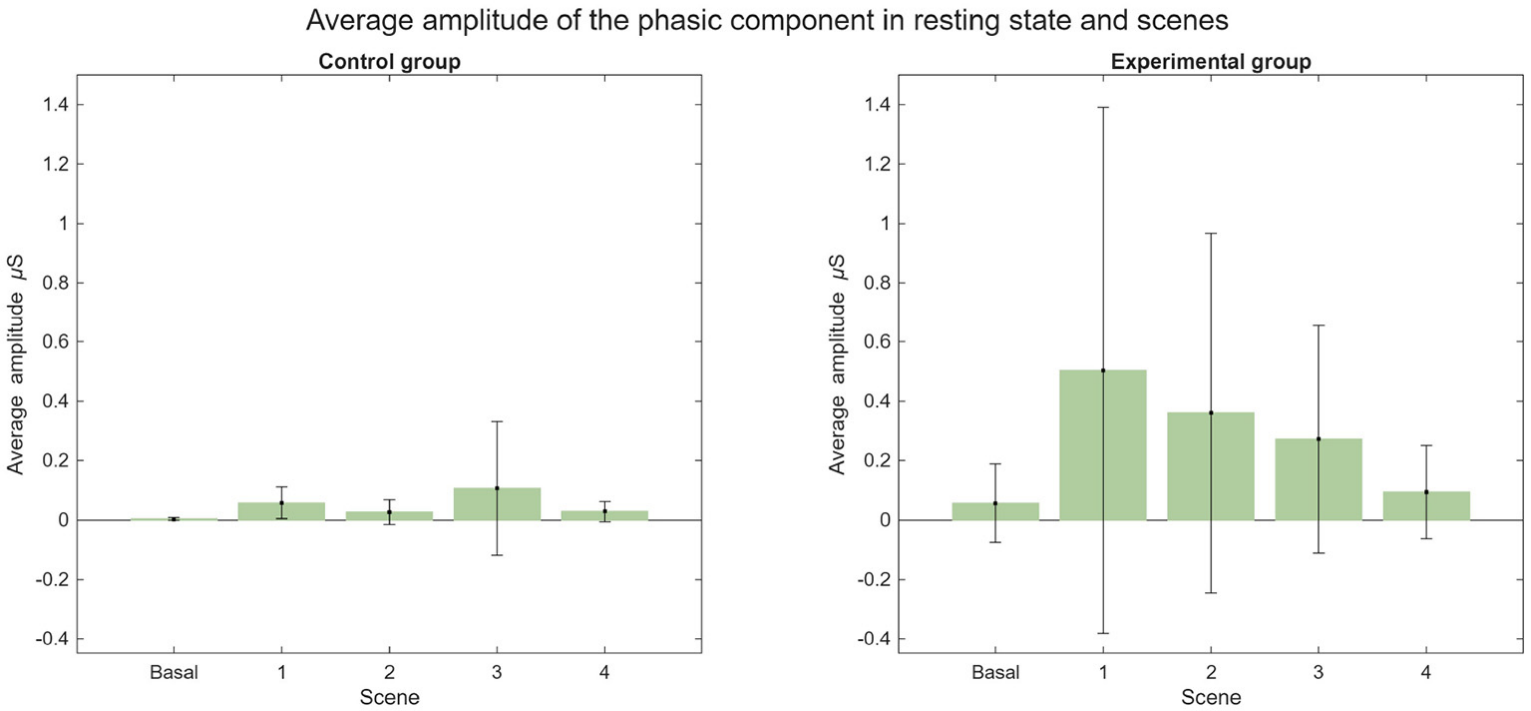
Average amplitude of the phasic component in resting state and scenes.

The average SCR for both groups during each scene is illustrated in Figure 19. Notably, two EDA recordings from the experimental group were lost due to data collection errors: subject 13 during Scene 1 and subject 23 during Scene 2. During the baseline, most subjects exhibited no SCRs, with the exception of four individuals. In the experimental group, two subjects registered a total of 13 SCRs, while in the control group, two subjects registered 1 and 4 SCRs, respectively. The low variability of EDA signals in a relaxed state likely explains the increased SCR frequency during experimental scenes compared to the baseline (Cosoli et al., 2021). Furthermore, higher SCR counts have been associated with high-level presence in specific environments (Moinnereau et al., 2022). Overall, a higher SCR count was observed in the experimental group, although both groups exhibited considerable standard deviation. The most prominent increases occurred during Scenes 1 and 3. In Scene 1, the control group averaged 9.5 ± 8.91 SCRs, while the experimental group averaged 12.75 ± 12.71. In Scene 3, the control group averaged 5.92 ± 7.76 compared to 11.25 ± 11.11 in the experimental group. The average phasic amplitude is presented in Figure 20. Notably, the experimental group exhibited a larger average amplitude than the control group across all scenes. The highest average phasic amplitude was recorded in the experimental group during Scene 1 (0.50 ± 0.89 μS). In contrast, the control group's largest average amplitude occurred during Scene 3 (0.11 ± 0.23 μS).

### 3.3 HR analysis

**Figure 21:**
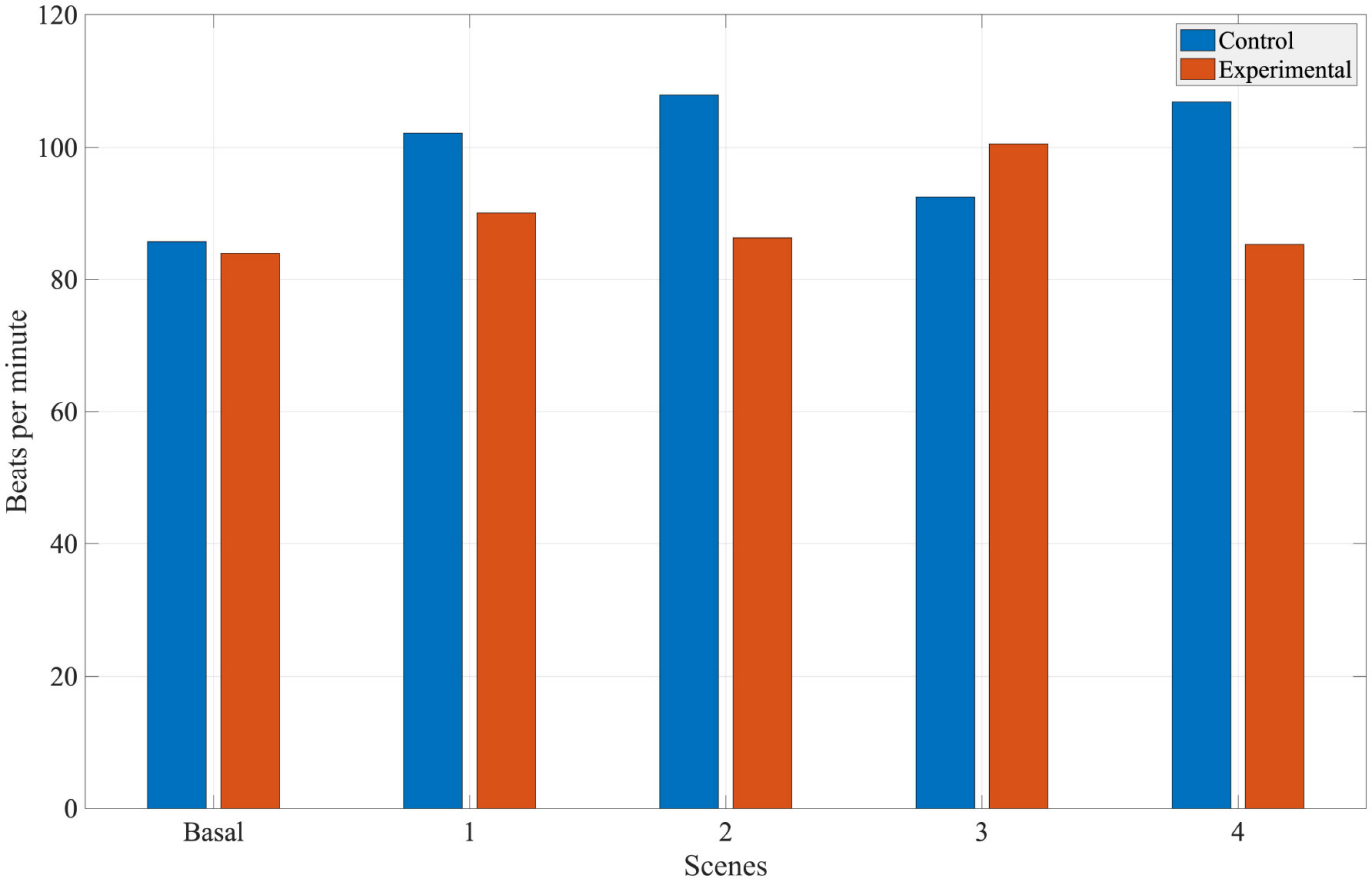
Average heart rate (beats per minute) across the four scenes comparing both groups.

As illustrated in Figure 21, the heart rate increased considerably more in the control group than in the experimental group. Existing literature suggests that a lower heart rate is associated with a greater sense of presence among participants (Moinnereau et al., 2022). While the heart rate did not drop below baseline values at any point, the relative difference between the two groups provides a basis for interpreting the results. The smaller increase in heart rate observed in the experimental group during Scenes 1, 2, and 4 suggests these participants experienced a higher level of presence than their counterparts in the control group (Grassini and Laumann, 2020). Specifically, tasks involving the expression of Descartes' passions, verbalizing passion-related words, and viewing paintings with facial recognition elicited a greater sense of presence than the control activities of abstract drawing, writing words, and manual painting analysis.

**Figure 22:**
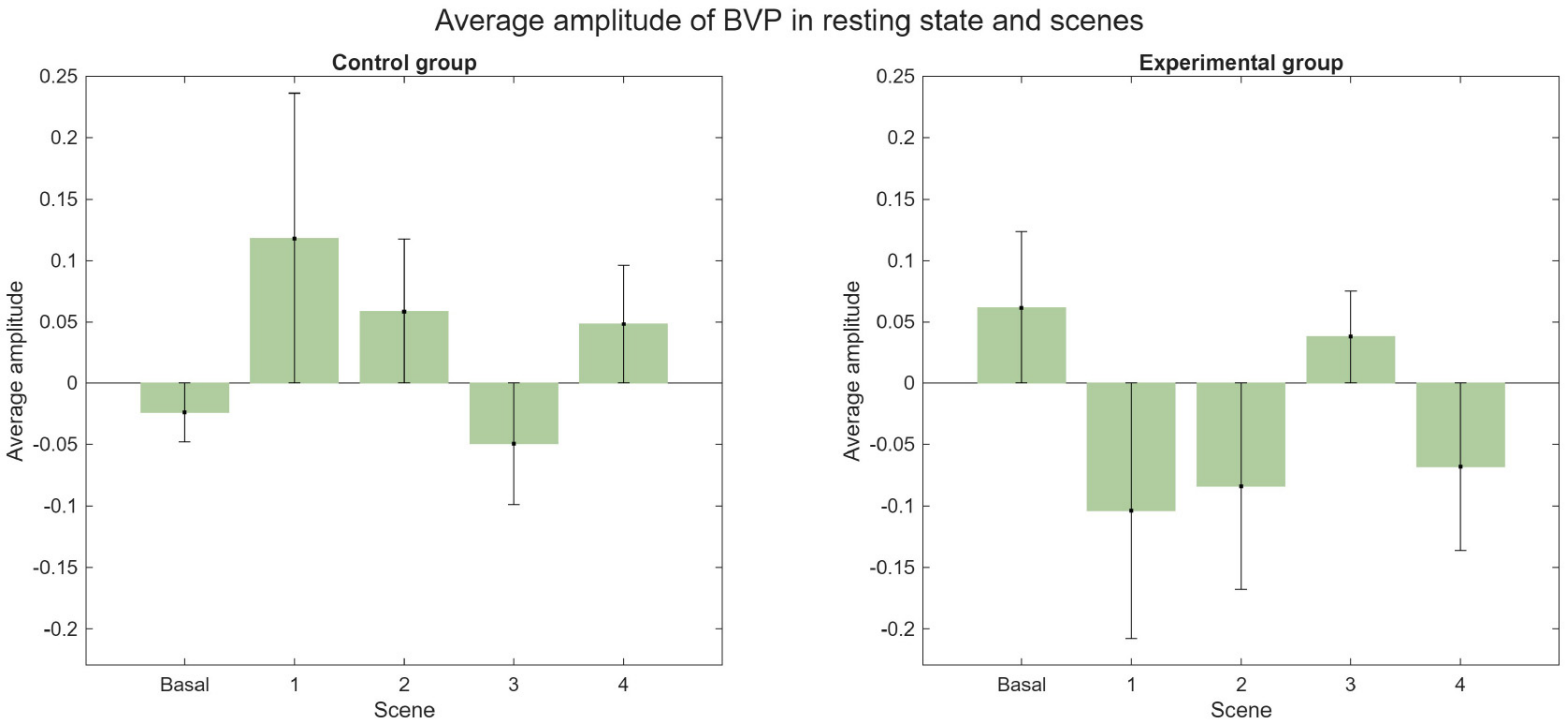
Average amplitude of BVP in resting state and scenes.

During Scene 3, participants in the control group demonstrated a higher level of presence than those in the experimental group. This suggests that reading literary quotes aloud and actively selecting words related to Descartes' passions elicited a greater sense of presence than the experimental condition, where participants read the quotes without a selection task. The average BVP amplitudes for each condition are illustrated in Figure 22.

### 3.4 Questionnaire statistical results

**Figure 23:**
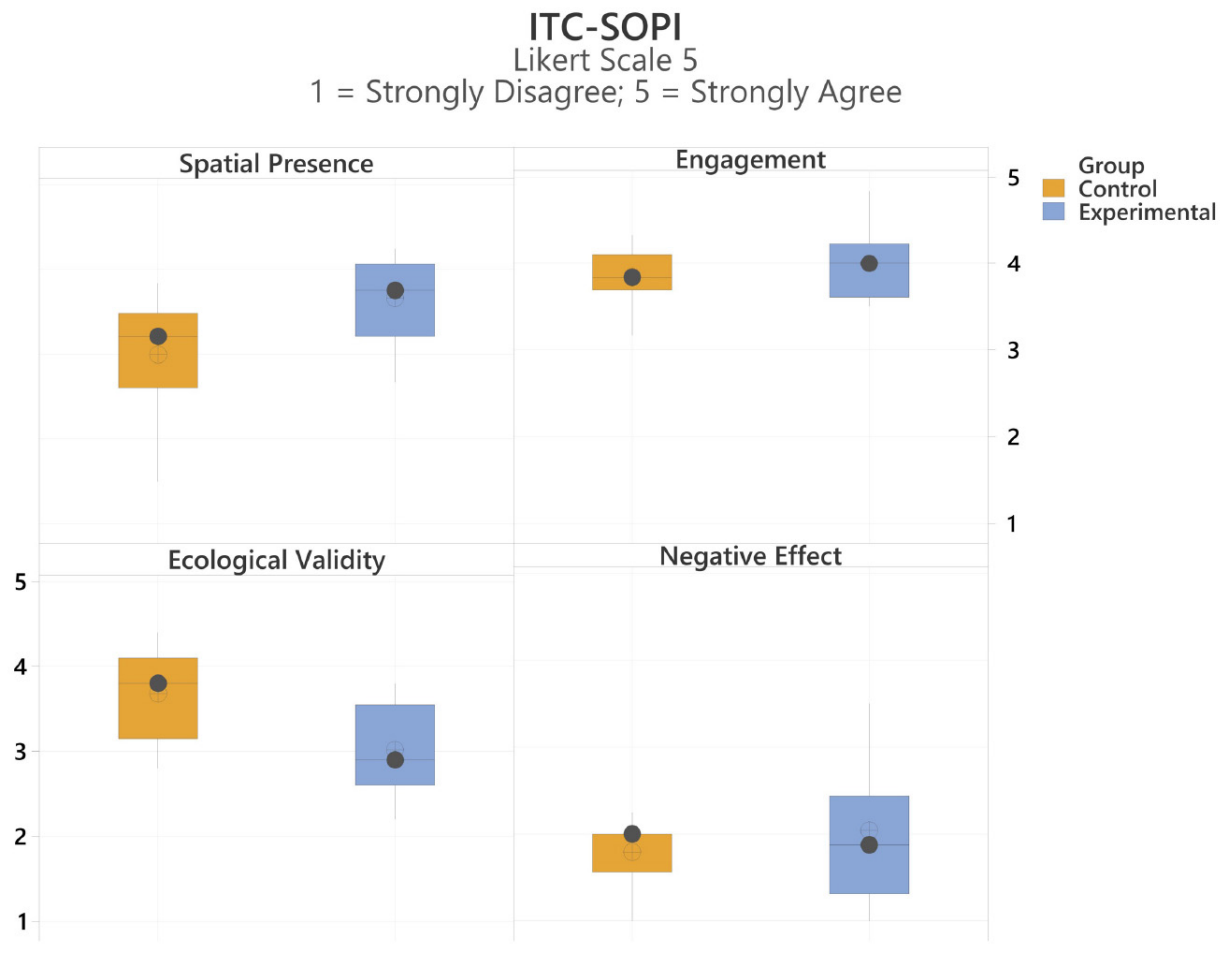
Box plot of statistical results from ITC-SOPI Presence Questionnaire. Likert scale was used (1 = strongly disagree, 5 = strongly agree).

The ITC-SOPI questionnaire was employed to collect qualitative data regarding the participants' sense of presence within the virtual environment. This instrument evaluates four distinct dimensions to compare various aspects of the user experience (Lessiter et al., 2001). Figure 23 presents the average scores obtained across each of these four categories.

Data normality for the ITC-SOPI dimensions was assessed using the Ryan-Joiner test in Minitab, which is particularly suitable for small sample sizes. The results indicated that all four dimensions were approximately normally distributed: Negative Effects (RJ = 0.991), Spatial Presence (RJ = 0.967), Engagement (RJ = 0.988), and Ecological Naturalness (RJ = 0.991). Based on these findings, parametric analyses were deemed appropriate. A one-way ANOVA was subsequently performed using a significance level of α = 0.05 to determine if significant differences existed between the groups. Table 5 presents the resulting *p*-values and eta-squared (η^2^) values, which measure the effect size and indicate the proportion of total variance attributable to the group effect. As defined by Becker (1999), η^2^ is calculated by dividing the effect variance by the total variance. The analysis revealed a significant effect of group on spatial presence, [*F*_(1, 22)_ = 7.63, *p* = 0.011], indicating that spatial presence differed significantly between the two conditions.

**Table 5:** Comparison of *p*-level and effect sizes with eta squared of the different categories of the ITC-SOPI questionnaire.

**Categories**	***p*-level**	**F-value**	***eta squared (h^2^)***
Spatial presence	0.011	7.63	0.257
Engagement	0.315	1.06	0.000
Ecological validity	0.005	9.53	0.302
Negative effects	0.344	0.93	0.041

Analysis of the ITC-SOPI categories revealed no significant differences between the two groups regarding engagement and negative effects. However, significant differences were observed in spatial presence and ecological validity. Specifically, spatial presence was significantly higher in the immersive group, whereas ecological validity was significantly lower compared to the control group. Notably, when considering effect sizes, ecological validity exhibited a stronger effect than spatial presence. According to the literature, lower *p*-values for spatial presence indicate a statistically significant and higher quality of immersion within the experimental setup. Conversely, higher scores in the ecological validity (naturalness) category reflect how realistically the experiment was perceived and how closely it resembles real-world experiences (Grassini and Laumann, 2020). These results suggest that while the experimental group experienced a higher quality of immersion and spatial presence, the lower ecological validity scores indicate the experience was perceived as innovative and unique rather than a direct simulation of everyday reality.

### 3.5 Content results

**Figure 24:**
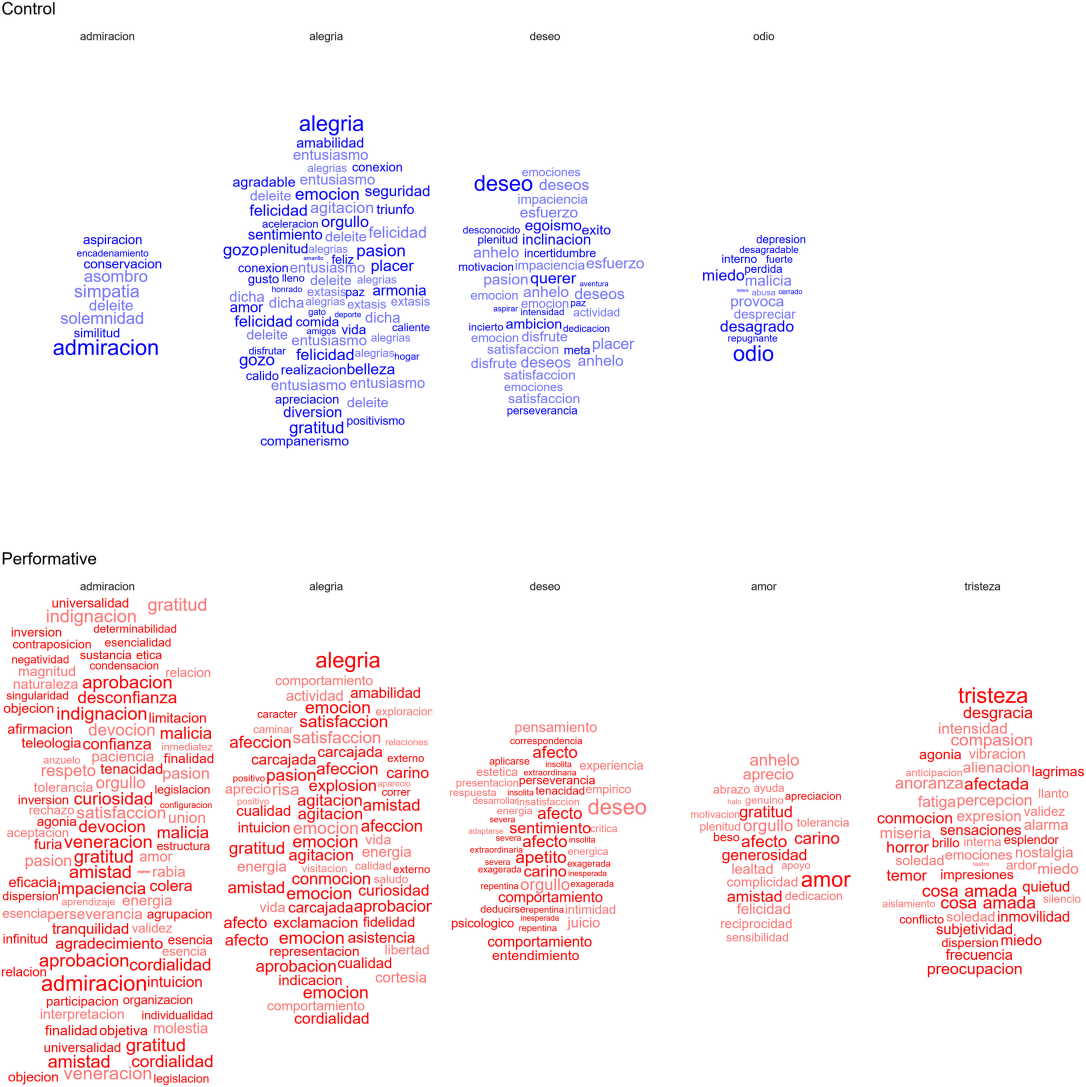
Proposed words (in Spanish) by selected passion. The size of the labels is related to the correlation that each word has with the corresponding passion within the corpus; the opaquer words were suggested by the experiment meanwhile the more transparent words were proposed by the participants.

In addition to neurophysiological measures, various strategies were implemented to evaluate educational impact beyond mere presence. During the second scene of both experiences, words proposed by each participant in relation to their experience and understanding of each studied passion were collected. The number of unique words suggested, along with their diversity in correlation with a specific corpus, serves as an indicator of the participants' vocabulary richness. This comparison is presented in Figure 24, where the number of words suggested by participants in the control group is contrasted with those in the immersive group. Regarding the words selected, a greater quantity and variety were observed in the immersive group compared to the control group. Furthermore, the selected words were more closely aligned with the specific passions chosen in each case; specifically, words were proposed in the immersive group that showed a higher correlation with the selected passion.

During scene four, the participants' reactions to a Vanitas artwork were analyzed. The number and variety of detected facial emotions served as indicators of the capacity to process diverse aesthetic reactions to the same painting. A greater diversity in captured emotions suggests an enhanced ability to appreciate and respond to complex artistic stimuli. Compared with the control group, a higher variety of emotions was experienced by the immersive group, with a mean of 5.5 emotions per participant compared to 2.5 emotions per participant in the control group. In the immersive group, the most frequently detected emotion was neutral, followed by fear, sadness, happiness, anger, surprise, and disgust. Similarly, for the control group, neutral was the most expressed emotion, followed by fear, surprise, happiness, anger, disgust, and sadness.

## 4 Limitations

Different limitations of this study should be acknowledged. First, the relatively small sample size may limit the generalization of the findings, particularly across different age groups. It is noted, however, that previous research has established heart rate and EEG signals as reliable indicators of engagement in virtual environments using a sample of 21 (Murphy and Higgins, 2019). Similarly, a significant positive impact of engagement on student outcomes was identified using a sample of 15 (Khedher et al., 2019). While these sample sizes are comparable to the one employed in the present study, a larger and more diverse participant pool is necessary for future research to further validate these findings. A significant drawback involved the loss of data from the Empatica E4 device, which necessitated the exclusion of three recordings from the experimental group. In future studies, strategies should be incorporated to minimize such data loss. Furthermore, while the experiment relied heavily on quantitative physiological signals, qualitative data were obtained via questionnaires, which may be susceptible to individual biases. Additionally, participants interacted with the environment only once, preventing conclusions regarding the long-term stability of the observed results. Further experiments involving repetitive use of the environment would be beneficial to determine if results remain consistent over continued exposure, particularly for therapeutic applications. A final limitation concerns the estimation of brain activations via dipole modeling, as the number of electrodes in the headset was relatively small (8). It is encouraged that readers consider the fact that a reduction in the number of electrodes decreases the accuracy of source location estimations (Soler et al., 2020; Bai and He, 2005). To compensate for this, participants were combined within their respective groups to enhance the accuracy of the estimations during cluster localization.

## 5 Conclusions

With the obtained results, it can be suggested that physiological signals such as EEG, EDA and HR may present a quantitative manner to measure the presence and engagement of a student within a partially immersive space. An increase in emotional activations, presence, and engagement was associated with participants in the partially immersive environment which shows that it could potentially be used as a technological tool to improve the learning experience and understanding of the Humanities. However, the field of study is broad and there are still limitations in the research that must be taken into account for future analysis.

Regarding EEG analysis, the calculation of features for the ML models supports the idea that the concepts of presence and engagement can be important factors in understanding the physiological changes of students in different educational environments. For instance, the ratio between beta over the sum of alpha and beta, related to engagement, and the behavior of the gamma band in the frontal electrodes were part of the features found with maximum relevance for classification. The selection of such features is in accordance to what has been previously mentioned in the literature, which confirms the cognitive processes that were present during the proposed experiences.

Furthermore, the high accuracy of the obtained ML models (close to 90%) may suggest that there is a notorious difference between the brain parameters that are present during the control, and immersive experiences.

From the dipole fitting analysis, it was observed that similar brain activations were found at both groups, specifically in the areas responsible for visual processing (10, 18 and 19), which goes hand in hand with the activities carried out during the experience. However, particular brain activations were found for specific Scenes and groups. For instance, the activation of brain areas related to somatosensory sensations (1 and 7) present in Scenes 1 and 3 from the experimental group. This activation may indicate the efficiency of the multisensory feedback provided in the partially immersive experience, which reflected the impact on the participants' reception of sensorial information and stimuli. Similarly, a cluster was observed in BA 40 during Scene 2 in the experimental group, which is related to emotional perception and processing. This results reflect the higher sensorial and emotional content in the immersive experience when compared to the more traditional experience.

Regarding the HR analysis, it was observed that there was less variability in the experimental group in terms of the basal state and the scenes, and that the results from the experimental group suggest a higher sense of presence. In the EDA analysis, the number of SCR peaks increased in both groups compared to their respective basal state. The average quantity of SCR peaks was very similar for both groups, noting that the average amount was slightly bigger in the control group. It is important to note, that there is great variability in the EDA signal between subjects, therefore there is great variation within both groups as shown by the high standard deviation. For further and more extensive research, it could be helpful to study the variation of EDA for each subject.

The results found from the analysis of the collected signals contribute preliminary evidence to support a better understanding of teaching in the Humanities and the effectiveness of the use of immersive technologies in learning. In the future, it is expected that the use of this first designed space can be scaled to a completely immersive version. Another interesting possibility is the implementation of the emotion detection algorithm into medical-clinical applications, for instance as assistive therapy tool for patients with mental disorders, or reduced emotional intelligence.

Nevertheless, ethical considerations in further uses such as full immersion and therapeutic uses must be taken into consideration. Although the study was conducted according to the Institution's ethics committee including informed consents, the data that can be obtained from further studies would be sensible and could be at risk of being manipulated, changing the main purpose of the project. Biometric data such as EEG signals, heart rate, movements and even emotions detected are personal, hence anonymization must be essential when publishing this data. Furthermore, if this data is used for medical applications such as therapy, data becomes more sensible, since disorders and diagnosis must be concatenated to the patient or user as well as their own data recorded. Misinterpretation or altering of these signals could eventually be harmful for patients. For this reason, biometric data for further studies regarding diagnosis and therapy must follow stricter regulations such as encryption, and controlled access.

## Data Availability

The datasets presented in this study can be found in online repositories. The names of the repository/repositories and accession number(s) can be found below: https://doi.org/10.6084/m9.figshare.24777084.
